# Phytochemical nanozymes reprogram redox for balanced antimicrobial and regenerative therapy in acute and chronic diabetic wounds

**DOI:** 10.1016/j.redox.2025.103718

**Published:** 2025-06-06

**Authors:** Yipeng Pang, Fructueux Modeste Amona, Xiaohan Chen, Yuxin You, Ziqi Sha, Zilu Liu, Jiamin Li, Yi Liu, Xingtang Fang, Xi Chen

**Affiliations:** a Institute of Cellular and Molecular Biology, School of Life Science, Jiangsu Normal University, Xuzhou, 221116, Jiangsu, China; b Department of Biophysics, School of Life Sciences, Xuzhou Medical University, Xuzhou, 221004, Jiangsu, China

**Keywords:** Phytochemical nanozymes, Redox modulation, Antimicrobial efficacy, Tissue regeneration, Diabetic wound healing

## Abstract

Chronic diabetic wounds are characterized by persistent oxidative stress and microbial infections, leading to delayed healing and tissue repair. While elevated reactive oxygen species (ROS) levels can provide bactericidal effects, uncontrolled oxidative stress simultaneously impairs tissue regeneration. Thus, precise redox modulation that balances antimicrobial efficacy with tissue regeneration is critical for effective wound therapy. Herein, we developed a phytochemical nanozymes system by integrating ferulic acid (FA) with cerium oxide nanoparticles (CeO_2_), enabling precise redox modulation to balance antimicrobial efficacy with tissue regeneration. Structural analysis confirmed the uniform dispersion and pH-responsive release of FA and Ce ions, facilitating targeted redox modulation. The FA-CeO_2_ nanozymes exhibited potent antioxidant activity through Ce^3+^/Ce^4+^ cycling and FA-mediated radical scavenging, effectively mitigating oxidative stress while promoting bacterial clearance against *S. aureus* and *E. coli*. Furthermore, FA-CeO_2_ significantly enhanced Nrf2/HO-1 pathway activation, leading to upregulated VEGF/CD31 expression, accelerated cell proliferation, and enhanced collagen deposition *in vitro*. *In vivo*, FA-CeO_2_ facilitated wound closure, reduced bacterial burden, and improved tissue regeneration in acute and diabetic wound models, with minimal cytotoxicity and excellent biocompatibility. These findings highlight the critical role of precise redox modulation in balancing antibacterial and regenerative therapy, positioning phytochemical nanozymes as a dual-modality platform for effective wound therapy and advancing nanomedicine strategies targeting oxidative stress and tissue repair.

## Graphical abstract

**Figure:**
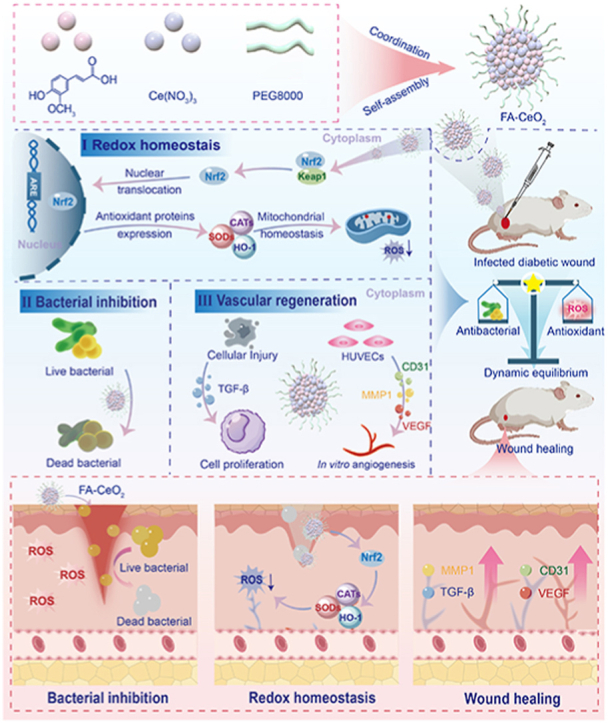

## 1 Introduction

Wounds are clinically sorted as either acute, healing within roughly 10 days, or chronic, which may require a longer timeframe owing to an intricate healing process [1,2]. Acute and chronic wounds, particularly in diabetic patients, are exacerbated by damaged blood vessels that increase bacterial infection susceptibility, inflammation, excessive generation of reactive oxygen species (ROS), and impair angiogenesis and tissue regeneration [3,4]. Furthermore, delayed re-epithelialization and lower skin tightening are key factors that impede wound closure in acute and diabetic chronic wounds [5]. Roughly 15–25 % of diabetics are at risk for chronic abscesses, potentially leading to amputations and posing substantial global health challenges [6,7].

ROS are crucial for tissue repair, as they are integral components of redox regulation in cellular signaling and pathogen defense [3]. However, persistent hyperglycemia in diabetes exacerbates ROS production owing to mitochondrial dysfunction [8], which fosters the proliferation of clinical bacterial pathogens like *Staphylococcus aureus* (*S. aureus)* in wounds [3,9]. While high ROS can exert bactericidal effects by disrupting microbial lipid/protein metabolism and respiratory pathways [10], excessive oxidative stress concurrently damages host tissues, exacerbating inflammation and delaying healing [3,11]. This dichotomy underscores the need for precise ROS modulation—balancing microbial control with tissue protection—to optimize wound repair, as highlighted in emerging redox-targeted therapies [11]. Henceforth, nanozyme systems capable of modulating ROS levels while retaining antimicrobial efficacy and tissue regeneration represent a promising strategy for diabetic wound management.

Recent advances have shifted focus on the new class of nanozymes mimicking enzymatic activities such as catalase (CAT), superoxide dismutase (SOD), and oxidase [12]. Unlike conventional ROS-dependent antimicrobials [3], these nanozymes minimize collateral oxidative damage to host tissues, driving research interest in multifunctional nanozyme platforms. NanoCeria (CeO_2_) has demonstrated significant antioxidant, anti-inflammatory, and enzyme-mimetic properties, making it a compelling candidate for wound healing applications [13,14]. However, the therapeutic potential of CeO_2_ can be further enhanced by incorporating bioactive phytochemicals, thus enabling synergistic redox modulation and antibacterial activity.

Phytochemicals have garnered substantial interest in nanomedicine due to their intrinsic antioxidant, anti-inflammatory, and antimicrobial properties [15,16]. Ferulic acid (FA), a phenolic compound characterized by its 4-hydroxy-3-methoxycinnamic acid structure, has been extensively studied for its antioxidative and tissue-regenerative properties [17]. In diabetic wound models, FA has been shown to enhance healing by upregulating growth factors (VEGF, PDGF), mitigating lipid peroxidation, and boosting endogenous antioxidants (CAT, SOD, GSH) [8,18]. Moreover, FA has demonstrated efficacy in reducing glycemic levels and promoting tissue regeneration, making it a promising therapeutic agent for diabetic wounds [19]. While FA and CeO_2_ have individually been explored for their roles in ROS modulation and wound healing, the integration of FA with CeO_2_ to form a phytochemical nanozyme system, specifically targeting acute and diabetic chronic wounds, remains unexplored. This knowledge gap is particularly relevant given the potential of such a dual-modality platform to synergistically regulate ROS, nuclear factor erythroid 2-related factor 2/Heme oxygenase-1 (Nrf2/HO-1) pathway, and promote antibacterial activity while facilitating tissue regeneration.

**Scheme 1:**
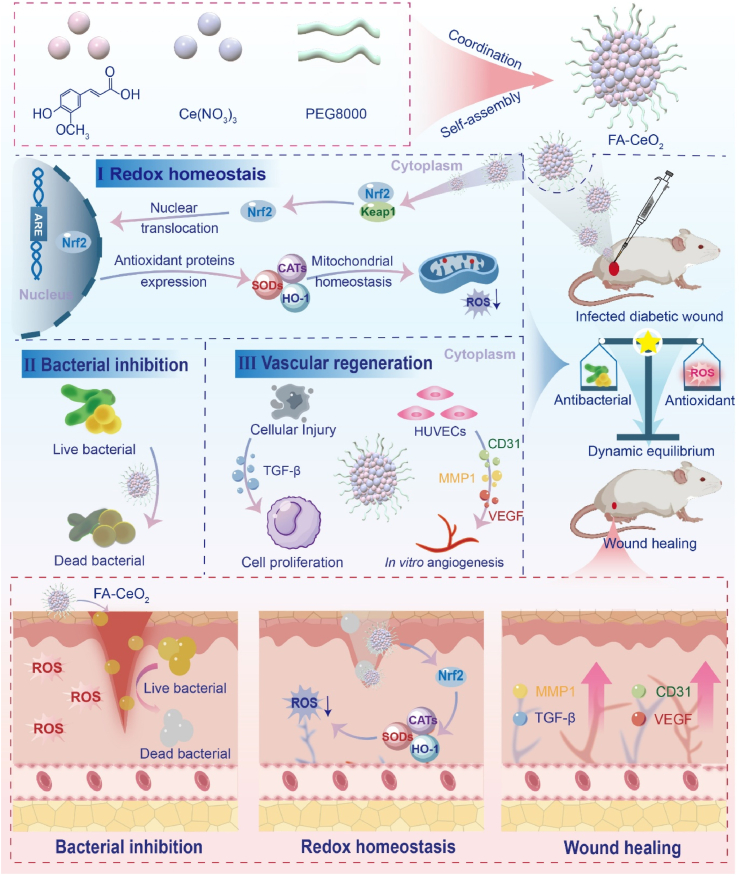
Schematic illustration of the precise redox regulation by phytochemical nanozymes (FA-CeO_2_) to balance antimicrobial and regenerative therapy in acute and diabetic chronic wound healing.

Hence, the core aim of this study was to evaluate the therapeutic potential of FA-CeO_2_ in acute and chronic diabetic wounds, focusing on ROS modulation, antibacterial activity, and tissue regeneration (Scheme 1). By integrating phytochemicals with nanozymes, this work aims to advance nanomedicine strategies not only for diabetic wounds but also for broader diseases related to oxidative stress, infection, and tissue regeneration.

## 2 Materials and methods

### 2.1 Reagents, materials, and bacterial strains

Cerium nitrate hexahydrate (Ce(NO_3_)_3_·6H_2_O, C105378), ferulic acid (F103701), PEG 8000 (P103734), hydrogen peroxide (H_2_O_2_, H112515), 1-Diphenyl-2-picrylhydrazyl (DPPH, D273092), and 2,2′-azino-bis (3-ethylbenzthiazoline-6-sulfonic acid) (ABTS, A109612) were sourced from Aladdin (Shanghai, China). Resazurin (R7017) and C11-BODIPY581/591 (SML3717) were obtained from Sigma-Aldrich (USA). Cell counting kit-8 (CCK-8, CA1210), 2′,7′-dichlorofluorescin diacetate (DCFH-DA, D6470), superoxide anion assay kit (BC1295), hydroxyl radical assay kit (BC1235), Calcein-AM/PI stain kit (CA1630), JC-1 mitochondrial membrane potential assay kit (M8650), and SOD/MDA/GPX/CAT determination kits (BC5165/BC0025/BC1195/BC0205) were purchased from Solarbio (Beijing, China). Crystal violet staining solution (C0121) and Live/Dead bacterial staining kit with DMAO & PI (C2030S) were obtained from Beyotime (Shanghai, China). RAW264.7 cells (CL-0190) and HUVECs cells (CP–H082) were purchased from Procell (China).

Antibodies against Nrf2 (16396-1-AP), NADPH oxidase 1 (NOX1, 17772-1-AP), HO-1 (10701-1-AP), SOD (10269-1-AP), Cluster of Differentiation 31 (CD31, 11265-1-AP), E-cadherin (20874-1-AP), Collagen I (14695-1-AP), Matrix Metalloproteinase 1 (MMP1, 10371-2-AP), Vascular endothelial growth factor (VEGF, 81323-2-RR) and transforming growth factor beta (TGF-β, 81746-2-RR), were acquired from Proteintech (Wuhan, China). Antibodies against glutathione peroxidase 4 (GPX4, ET1706-45), GAPDH (R1210-1), and Tubulin (M0805-8) were sourced from HUABIO (Hangzhou, China).

The strains of *Staphylococcus aureus* (*S. aureus*, ATCC 25923) and *Escherichia coli* (*E. coli*, ATCC 25922) were obtained from Fuxiang (Shanghai, China).

### 2.2 Preparation and synthesis of FA-CeO_2_

FA-CeO_2_ nanoparticles were prepared using a one-pot synthesis protocol, modified from an established procedure [20]. PEG 8000 (20 mg mL^−1^) and Ce(NO_3_)_3_·6H_2_O (47.45 mg mL^−1^) were separately dissolved in ultrapure water and then combined in a glass reaction vessel in a stepwise manner. Ferulic acid (FA, 5 mg mL^−1^) was subsequently incorporated into the mixture to trigger the self-assembly phase. The reaction was maintained under ambient conditions with continuous stirring for 20 min to ensure the formation of stable FA-CeO_2_ nanostructures. Residual unreacted precursors or impurities were eliminated via centrifugation at 8000×*g* for 10 min. Finally, the purified nanoparticles were subjected to lyophilization, yielding a dry, free-flowing powder for subsequent analytical characterization.

### 2.3 Characterization of FA-CeO_2_

A suite of analytical techniques was used to characterize the FA-CeO_2_ nanoparticles. High-resolution surface morphology and elemental distribution imaging were achieved using scanning electron microscopy (SEM; Zeiss Sigma 300) and transmission electron microscopy (TEM; JEOL F200). The chemical identity of CeO_2_ was confirmed via X-ray photoelectron spectroscopy (XPS) on a Thermo Fisher Scientific K-Alpha system. At the same time, its crystalline structure was analyzed by X-ray diffraction (XRD) using a Bruker D8 Advance instrument. Optical and molecular properties were investigated through ultraviolet–visible (UV–Vis) spectroscopy and Fourier-transform infrared (FTIR) spectrometry. Additionally, dynamic light scattering (DLS) was applied to measure hydrodynamic diameter and size distribution, with colloidal stability evaluated via zeta potential measurements conducted on a Malvern ZetaSizer Nano ZS. This comprehensive approach ensured detailed physicochemical profiling of the nanoparticles.

Furthermore, the release of cerium (Ce^3+/4+^) ions and FA from FA-CeO_2_ composites was monitored at specific intervals. After separating the mixture via centrifugation, the supernatant was analyzed using UV–vis spectrophotometry, with Ce ions and FA concentrations determined through a standard calibration curve protocol.

### 2.4 Stability of FA-CeO_2_ in different solvents

The colloidal stability of FA-CeO_2_ nanoparticles was evaluated by suspending the material in deionized water, 0.9 % NaCl, phosphate-buffered saline (PBS), and Dulbecco's Modified Eagle Medium (DMEM) at a concentration of 0.5 mg mL^−1^. The mixture was homogenized via 15-min ultrasonication, and particle size dynamics were tracked over 168 h using DLS to detect agglomeration or sedimentation. The suspensions were stored under refrigerated (4 °C) conditions to evaluate long-term stability, with observations recorded over extended durations.

### 2.5 ROS scavenging experiments

#### 2.5.1 ABTS and DPPH radicals scavenging activity of FA-CeO_2_

The direct antioxidant capacity was evaluated by measuring the ABTS and DPPH radical scavenging activity of FA-CeO_2_. A 7 mM ABTS aqueous solution and 2.45 mM potassium persulfate were prepared and incubated under dark conditions at ambient temperature for 24 h to obtain ABTS radical cations (ABTS•^+^), as previously described [20,21]. The resulting ABTS•^+^ solution was then treated with varying concentrations of FA-CeO_2_ for 10 min. Absorbance measurements at 734 nm were conducted via UV–vis spectroscopy to assess antioxidant activity. Furthermore, for DPPH radical (DPPH•) analysis, a 125 μmol mL^−1^ DPPH• solution was combined with FA-CeO_2_ at a 1:1 vol ratio and incubated for 10 min at 37 °C. The absorbance at 517 nm was subsequently recorded using UV–vis spectroscopy to quantify DPPH scavenging capacity [20].

#### 2.5.2 •OH and H_2_O_2_ radicals scavenging activity of FA-CeO_2_

Hydroxyl radicals (•OH) were generated via a Fenton reaction system containing 100 μL of 32 mM FeCl_3_·6H_2_O, 5 % hydroxylamine hydrochloride, and 100 μL of 300 mM H_2_O_2_. Methylene blue trihydrate (MB, 100 μL, 60 μg/mL) was employed to trap the •OH radicals. Different concentrations of FA-CeO_2_ were mixed into the •OH-containing solution, and spectrophotometric analysis at 664 nm was performed to evaluate the •OH scavenging efficiency of FA-CeO_2_.

To assess H_2_O_2_ scavenging activity, a commercial H_2_O_2_ detection assay was utilized. H_2_O_2_ reacts with ammonium molybdate to produce a stable yellow-colored complex with maximum absorbance at 405 nm. FA-CeO_2_ at different concentrations was incubated with 2 mM H_2_O_2_ at 37 °C for 24 h. After incubation, residual H_2_O_2_ levels were quantified according to the kit protocol, enabling the calculation of FA-CeO_2_'s H_2_O_2_ elimination capacity.

### 2.6 Bacteria experiments analysis

#### 2.6.1 Bacteria count assay

Bacterial viability was analyzed using a colony-forming unit (CFU) quantification method to evaluate the antimicrobial effects of FA, Ce ion, and FA-CeO_2_. All treatment groups were administered at equivalent active component concentrations, with FA (51.2 μg/mL) and Ce ion (35.4 μg/mL) matched to their respective contents in FA-CeO_2_ (128 μg/mL), to evaluate the FA-CeO_2_ 's antimicrobial advantage over free compounds. Post-treatment bacterial suspensions were standardized to 1 × 10^8^ CFU/mL, inoculated onto nutrient agar plates, and cultured at 37 °C for 18–24 h. Colony growth was visually documented, and enumerating CFUs quantified bacterial survival.

#### 2.6.2 Resazurin staining test

Bacterial metabolic activity post-FA (51.2 μg/mL), Ce ion (35.4 μg/mL), and FA-CeO_2_ (128 μg/mL) exposure was analyzed using a resazurin-based viability assay. Treated bacterial cultures in 96-well plates were incubated with resazurin solution at 37 °C for 4 h. A shift in solution color from blue (oxidized state) to pink (reduced state) was observed, correlating with bacterial metabolic function. The intensity of the pink coloration served as an indicator of viability, with stronger hues denoting higher survival rates and diminished changes suggesting the antibacterial efficacy of FA-CeO_2_.

#### 2.6.3 Investigation of bacterial morphology

Bacterial cultures were grown for 24 h and then incubated with FA (51.2 μg/mL), Ce ion (35.4 μg/mL), and FA-CeO_2_ (128 μg/mL) for 2 h. Following treatment, the samples were stabilized using 2.5 % glutaraldehyde at 4 °C for 3 h. Afterward, the bacterial cells underwent three washes with sterile PBS and sequential dehydration using increasing ethanol concentrations. The dehydrated bacteria were freeze-dried and coated with a conductive metal layer before SEM analysis. Morphological alterations in bacterial cells were examined using SEM [22].

#### 2.6.4 Live/dead bacterial staining test

Bacterial viability following FA (51.2 μg/mL), Ce ion (35.4 μg/mL), and FA-CeO_2_ (128 μg/mL) exposure was assessed via a Live/Dead Bacterial Viability Kit (DMAO/PI) per the supplier's guidelines. Treated bacterial cells were harvested via centrifugation, washed, and reconstituted in PBS. A dual-stain solution was prepared by combining DMAO (viable cell indicator) and propidium iodide (PI, dead cell marker) at specified ratios. The bacterial suspension was then treated with the stain mixture and incubated under dark conditions at ambient temperature for 15 min. Fluorescence microscopy was employed to observe the appearance of the bacteria, with ImageJ software (National Institutes of Health, USA) used to quantify fluorescence signals and derive viability ratios.

#### 2.6.5 Bacterial biofilm biomass detection

Crystal violet staining protocol was employed to evaluate the antimicrobial effect of FA (51.2 μg/mL), Ce ion (35.4 μg/mL), and FA-CeO_2_ (128 μg/mL) on biofilm formation. Bacterial cultures were seeded into 24-well plates and incubated at 37 °C for 24 h under static conditions to facilitate biofilm formation. After incubation, non-adherent cells were removed by gently rinsing the wells with PBS. Adherent biofilms were fixed and treated with 0.1 % crystal violet for 15 min at ambient temperature. The unbound stain was eliminated via PBS washing, and biofilm-associated dye was dissolved using a 95 % ethanol solution. Absorbance measurements of the solubilized crystal violet were performed at 595 nm, and biofilm quantification was conducted using ImageJ.

### 2.7 Cell experiments analysis

#### 2.7.1 Cellular viability assay

To assess the cytotoxic effects of FA-CeO_2_, RAW264.7 cells and human umbilical vein endothelial cells (HUVECs) were cultured in 96-well plates at a density of 1 × 10^4^ cells/well in 100 μL DMEM supplemented with fetal bovine serum (FBS) and antibiotics (penicillin and streptomycin). Cells were exposed to FA (12.8 μg/mL), Ce ion (8.85 μg/mL), and FA-CeO_2_ (32 μg/mL) and maintained under standard culture conditions (37 °C, 5 % CO_2_) for 24 h. Post-treatment, cellular viability was quantified using a Cell Counting Kit-8 (CCK-8) colorimetric assay [21].

#### 2.7.2 Live/dead assay of cells

RAW264.7 cells and HUVECs cells were seeded at a density of 1 × 10^6^ cells per well in 6-well plates and allowed to adhere overnight. FA (12.8 μg/mL), Ce ion (8.85 μg/mL), and FA-CeO_2_ (32 μg/mL) were added to the wells for a 2-h pretreatment. Subsequently, the cells were stimulated with H_2_O_2_ (final concentration: 400 μM) for 24 h. The cells were stained with calcein-AM (for live cells) and propidium iodide (for dead cells). After a 20-min incubation, the cells were washed with PBS. Finally, fluorescence images were captured using microscopy (Olympus, Japan).

#### 2.7.3 Intracellular ROS detection

RAW264.7 cells were pretreated for 2 h with FA (12.8 μg/mL), Ce ion (8.85 μg/mL), and FA-CeO_2_ (32 μg/mL). Following pretreatment, ROS induction was triggered by exposing the cells to 400 μM H_2_O_2_ for 24 h. The fluorescent probe DCFH-DA was then added and incubated at 37 °C under dark conditions for 30 min. Fluorescence signals were visualized using fluorescence microscopy (Olympus, Japan), and ROS levels were quantified via ImageJ.

#### 2.7.4 Mitochondrial membrane potential assay

Mitochondrial membrane potential (ΔΨm) was analyzed using the fluorescent cationic dye JC-1. RAW264.7 cells were pretreated for 2 h with FA (12.8 μg/mL), Ce ion (8.85 μg/mL), and FA-CeO_2_ (32 μg/mL). Oxidative stress was induced by exposing the cells to 400 μM H_2_O_2_ for 24 h. After treatment, cells were incubated with JC-1 (5 μg/mL) at 37 °C for 20 min. Fluorescence microscopy (Olympus, Japan) was used to capture images of JC-1 aggregates (red fluorescence, intact ΔΨm) and monomers (green fluorescence, depolarized mitochondria). Fluorescence intensity ratios (red/green) were quantified using ImageJ software to assess mitochondrial potential [13,23,24].

#### 2.7.5 Detection of lipid peroxidation levels

Lipid peroxidation levels in RAW264.7 cells were evaluated using the fluorescent probe C11-BODIPY (Sigma-Aldrich, USA). Post-treatment with FA (12.8 μg/mL), Ce ion (8.85 μg/mL), and FA-CeO_2_ (32 μg/mL), cells were incubated with 2 μM C11-BODIPY in serum-free DMEM for 30 min at 37 °C under dark conditions. An unbound probe was removed by washing it twice with PBS. Fluorescence microscopy (Olympus, Japan) was employed to visualize lipid peroxidation, with non-oxidized lipids emitting green fluorescence (excitation: 488 nm) and oxidized lipids fluorescing red (excitation: 590 nm). ImageJ software was used to quantify fluorescence intensities, and the ratio of red-to-green signal intensity was calculated to determine peroxidation levels.

#### 2.7.6 Evaluation of antioxidant activity in RAW264.7 cells

Post-treatment with FA (12.8 μg/mL), Ce ion (8.85 μg/mL), and FA-CeO_2_ (32 μg/mL), cellular proteins were isolated using radioimmunoprecipitation assay (RIPA) lysis buffer, and total protein concentration was quantified via a bicinchoninic acid (BCA) assay following the supplier's protocol. Oxidative stress markers, including catalase (CAT), superoxide dismutase (SOD), malondialdehyde (MDA), and glutathione peroxidase (GPx), were analyzed in the cell lysate supernatant. Enzyme-specific activity assays were performed using commercially available kits, with each biomarker's levels determined by the corresponding manufacturer's guidelines.

#### 2.7.7 EdU assay for cell proliferation

BeyoClick™ EdU Cell Proliferation Kit (Beyotime, Shanghai, China) was used to evaluate the proliferative activity of treated HUVECs. Post-treatment with FA (12.8 μg/mL), Ce ion (8.85 μg/mL), and FA-CeO_2_ (32 μg/mL), the cells were exposed to EdU solution for 2 h. The samples were then rinsed with PBS, fixed, and counterstained with DAPI solution to label the nuclei. Fluorescence imaging was performed using an inverted microscope (Olympus, Japan), and the ratio of EdU-positive cells was quantified with ImageJ.

#### 2.7.8 Scratch assay for cell migration

The migratory capacity of treated HUVECs was evaluated through a scratch wound healing assay. HUVECs were cultured in 6-well plates until reaching full confluency. A scraped line was created in the cell monolayer using a sterile 200 μL pipette tip. Following PBS rinsing to eliminate dislodged cells, fresh medium containing different treatments (FA-CeO_2_ at 32 μg/mL, FA at 12.8 μg/mL, and Ce ions at 8.85 μg/mL) was added to each group. Migratory progression was tracked at 0, 24, and 48 h post-scratch. Phase-contrast microscopy (Olympus, Japan) was used to capture images of the wound area, and ImageJ software was utilized for computational quantification of cell migration by measuring the reduction in wound area over time [21].

#### 2.7.9 Tube formation assay for cell angiogenic capability

Endothelial tube formation was evaluated via a Matrigel-based angiogenesis assay. A 24-well plate was pre-coated with 50 μL of Matrigel matrix (Corning, NY, USA) and allowed to polymerize at 37 °C for 30 min. Cells were then seeded onto the matrix and cultured for 12 h under standard conditions. Following the post-treatment with FA (12.8 μg/mL), Ce ion (8.85 μg/mL), and FA-CeO_2_ (32 μg/mL), tubular network formation was examined using phase-contrast microscopy (Olympus, Japan), with total tubular length and branch points quantified computationally using ImageJ [25].

#### 2.7.10 Transwell migration assay

Cell migratory potential was analyzed using a Transwell chamber system. Briefly, 2 × 10^4^ cells were plated in the upper compartment of 8 μm pore polycarbonate Transwell inserts (Corning, NY, USA) housed in a 24-well plate. The lower chambers were loaded with 600 μL of DMEM supplemented with 10 % FBS, while the upper compartments contained 200 μL of serum-free medium. Cells were treated with FA (12.8 μg/mL), Ce ion (8.85 μg/mL), or FA-CeO_2_ (32 μg/mL) for 24 h. Following the incubation at 37 °C, non-migratory cells remaining on the apical membrane surface were kindly removed using cotton swabs. Migrated cells adherent to the basal membrane were immobilized with 4 % paraformaldehyde, stained with 1 % crystal violet, and rinsed with PBS. Cellular migration was documented via inverted microscopy (Olympus, Japan), with subsequent quantification of traversed cells performed using ImageJ [26].

### 2.8 Hemolysis assay

Hemolytic activity was performed as previously described [20]. Mouse erythrocytes were isolated by centrifuging 0.5 mL of whole blood (3500 rpm for 5 min) and washed three times with sodium thiobarbital solution to prepare a 5 % (v/v) erythrocyte suspension. Serial dilutions of FA-CeO_2_ (32, 64, 128, 256, 512 μg/mL) were combined with the erythrocyte suspension and agitated (100 rpm) at 37 °C for 1 h. Post-incubation, samples were centrifuged (3500 rpm, 5 min), and the absorbance of the supernatant was measured spectrophotometrically at 545 nm. PBS and deionized water served as negative and positive controls, respectively.

The hemolysis ratio was calculated using the formula:

$$Hemolysis\;ratio\;\left(\%\right)=\frac{{\text{OD}}_{\text{sample}}-{\text{OD}}_{\text{PBS}}}{{\text{OD}}_{\text{water}}-{\text{OD}}_{\text{PBS}}}\times 100\%$$

OD_sample_ represents the absorbance of erythrocytes exposed to different concentrations of FA-CeO_2_, respectively.

### 2.9 Acute full-thickness cutaneous wound healing assessment

Animal experiments complied with the National Institutes of Health's Guide for the Care and Use of Laboratory Animals and were approved by the Animal Ethics Committee of Jiangsu Normal University (Assigned Protocol Number: JSNU-IACUC 2025026). All bacterial infection experiments, including applying *S. aureus* to diabetic wounds, were conducted following institutional biosafety protocols. The work was performed in a biosafety level 2 (BSL-2) facility, which is appropriate for handling *S. aureus* and other opportunistic pathogens. Personnel followed all relevant biosafety practices, including the use of personal protective equipment (PPE), proper sterilization of materials, and disposal of biological waste.

Twenty-four adult male ICR mice (6–8 weeks old, 25–30 g) were acclimatized in polypropylene cages under controlled environmental conditions (25 °C, 12-h light/dark cycle) with ad libitum access to food and water. To minimize hormonal variability, such as estrogen fluctuations in females that could confound wound healing outcomes, male mice were selected based on established protocols in diabetic wound studies [13,14]. Mice were randomly allocated into four experimental cohorts (n = 6/group): Group 1 (PBS control), Group 2 (FA), Group 3 (Ce ions), and Group 4 (FA-CeO_2_). Full-thickness cutaneous wounds (8 mm diameter) were surgically excised using a biopsy punch and inoculated with *S. aureus* for 72 h to establish infection. Bacterial colonization was verified through macroscopic observation of purulent exudate (photographically documented) and microbial culture. FA (0.4 mg/kg), Ce ion (0.277 mg/kg), FA-CeO_2_ (1 mg/kg), or PBS (control) were administered topically once daily for 7 days. Therapeutic outcomes were evaluated via longitudinal monitoring of body weights and wound dimensions. Post-treatment, mice were humanely euthanized, and infected wound tissues, alongside major organs (heart, liver, spleen, lungs, kidneys), were harvested and cryopreserved at −80 °C. Wound tissues from three mice per group were subsampled for protein and gene expression profiling (Western blotting, RT-qPCR), while residual specimens underwent histological evaluation.

### 2.10 Assessment of chronic wound healing in STZ-induced diabetic mice

Twenty-four adult male ICR mice (6–8 weeks old) were acclimatized to laboratory conditions for 7 days pre-experiment. Before streptozotocin (STZ) administration, animals underwent a 12-h fasting period with ad libitum water access. Diabetes was induced via a single intraperitoneal (*i.p.*) injection of freshly reconstituted STZ (50 mg/kg body weight) dissolved in 0.1 M citrate buffer (pH 4.5). Post-injection, unrestricted access to food and water was reinstated. Longitudinal blood glucose monitoring was performed on days 3 and 7 post-STZ administration using a OneTouch Ultra glucometer via tail vein sampling. Mice exhibiting sustained hyperglycemia (>16.7 mmol/L or 300 mg/dL) across two consecutive measurements were confirmed as diabetic and advanced to subsequent experimental phases [8]. After confirming the diabetic status, full-thickness cutaneous wounds (∼8 mm in diameter) were created on the mid-back using a sterile biopsy punch. To establish infection, 20 μL of a *S. aureus* suspension (1 × 10^8^ CFU/mL) was topically applied to the wound bed immediately after injury for 72 h. The wound was then left uncovered to simulate a chronic wound environment. Mice were monitored daily for general health, wound appearance, and infection signs. Subsequently, FA (0.4 mg/kg), Ce ion (0.277 mg/kg), FA-CeO_2_ (1 mg/kg), or PBS (control) were administered topically once daily for 10 days. Therapeutic outcomes were evaluated via longitudinal monitoring of body weights and wound dimensions. Post-treatment, mice were humanely euthanized, and infected wound tissues, alongside major organs (heart, liver, spleen, lungs, kidneys), were harvested and cryopreserved at −80 °C. Wound tissues from three mice per group were subsampled for protein and gene expression profiling (Western blotting, RT-qPCR), while residual specimens underwent histological evaluation.

The wound contraction rate was evaluated using the following equation:

$$Wound\;contraction\;\left(\%\right)=\frac{\left(C_{0}-C_{n}\right)}{C_{0}}\times 100\;\%$$

C_0_ and C_n_ represent the initial wound area at different time points, respectively.

### 2.11 Bacterial survival assessment in wounds

Wound tissue specimens were aseptically excised from treatment groups after FA-CeO_2_ administration, using sterile surgical blades and immersed in PBS. Tissues were homogenized by vortex mixing (5 min) to release bacterial pathogens. Serial dilutions of the homogenates were plated on Müller-Hinton agar (MHA) and incubated aerobically at 37 °C for 24 h. Bacterial colonization was enumerated via CFU counts to assess the antimicrobial efficacy of FA-CeO_2_ in reducing wound bioburden, as previously described [27].

### 2.12 Histology and immunohistochemistry staining analysis

Excised wound tissues were fixed in 4 % paraformaldehyde, dehydrated, and paraffin-embedded. Sections (4 μm thickness) were prepared using a rotary microtome, dewaxed, and subjected to hematoxylin-eosin (H&E) and Masson's trichrome staining to visualize tissue architecture and collagen deposition, respectively [14]. Stained sections were analyzed using bright-field microscopy (Leica, Germany) to document morphological and fibrotic changes. Parallel histological assessments of major organs (heart, liver, spleen, lungs, kidneys) were conducted using identical processing protocols to evaluate systemic biocompatibility [13].

For immunohistochemistry (IHC), tissue sections underwent permeabilization with 0.3 % Triton X-100 for 10 min, followed by blocking in 5 % goat serum at ambient temperature (30 min). Primary antibodies targeting Nrf2 and HO-1 (diluted in BSA buffer, 1:200) were applied to the sections, which were then incubated overnight at 4 °C. After three washes with PBS, sections were treated with fluorophore-conjugated secondary antibodies for 1 h at room temperature. Nuclear counterstaining was performed using DAPI, and fluorescent signals were visualized using an optical microscope (Olympus, Tokyo, Japan). Quantitative analysis of IHC staining intensity was conducted using ImageJ.

### 2.13 Western blotting analysis

Protein samples were resolved through 10 % SDS-polyacrylamide gel electrophoresis (SDS-PAGE) and electrophoretically transferred onto cellulose nitrate membranes. Nonspecific binding sites were blocked with 5 % nonfat dry milk in Tris-buffered saline for 2 h at ambient temperature. Membranes were probed with primary antibodies (diluted in blocking buffer, 1:1000) overnight at 4 °C, followed by incubation with horseradish peroxidase (HRP)-conjugated secondary antibodies. Protein bands were visualized using an enhanced chemiluminescence (ECL) detection reagent (Biosharp, Hefei, China) and imaged with a chemiluminescence documentation system (Tanon, Shanghai, China). GAPDH expression served as the loading control for normalization, and quantitative densitometric analysis was performed using ImageJ.

### 2.14 Immunofluorescence assay

Wound tissue sections from mice were air-dried and immobilized in 4 % paraformaldehyde (PFA) for 10 min at ambient temperature. Following three sequential 5-min rinses with PBS, sections were blocked with 5 % goat serum diluted in PBS for 1 h. Primary antibodies targeting NOX1, GPX4, CD31, and VEGF (diluted in BSA buffer, 1:200) were applied and incubated overnight at 4 °C. After PBS washes, fluorophore-conjugated secondary antibodies were added for 2 h at room temperature. Nuclei were counterstained with DAPI for 10 min, and fluorescence signals were documented using an Olympus fluorescence microscopy system. ImageJ was employed for quantitative analysis of fluorescence intensity [13].

### 2.15 Quantitative real-time (qRT) PCR

RNA extraction from wound tissues was performed using the RNeasy Mini Kit (Qiagen, Germany), followed by cDNA synthesis with a reverse transcription PCR kit (TaKaRa, Japan). Gene expression analysis was carried out via qRT-PCR using SYBR Premix Ex Taq™ II (Tli RNaseH Plus; TaKaRa). Transcript levels of target genes were quantified via the 2^−ΔΔCt^ method, with GAPDH serving as the endogenous reference for normalization. Data were expressed relative to the control group. Primer pair sequences used in this study are detailed in Supplementary Table S1.

### 2.16 Blood biochemical analysis

A serological evaluation was conducted to monitor hepatic and renal function biomarkers to assess the systemic safety potential of FA-CeO_2_ nanocomposites. Following administration of FA-CeO_2_ (4 mg/kg), blood samples were collected from treated mice. Serum was separated via centrifugation (3000 rpm, 10 min, 4 °C) and analyzed for alanine aminotransferase (ALT), aspartate aminotransferase (AST), blood urea nitrogen (BUN), and creatinine (CRE) levels. These parameters were quantified using standardized commercial assay kits (Nanjing Jiancheng, China) according to the manufacturer's instructions.

### 2.17 Statistical analysis

Statistical analyses were conducted using SPSS (v21.0, IBM, USA), with results reported as mean ± standard deviation (SD). Graphical data representations were generated using GraphPad Prism 8.3 (San Diego, CA, USA). Intergroup comparisons were performed via one-way analysis of variance (ANOVA) with Tukey's post-hoc testing, adopting a significance threshold of *p < 0.05, p < 0.01, and p < 0.001*.

## 3 Results and discussion

### 3.1 Preparation and characterization of FA-CeO_2_

**Fig. 1:**
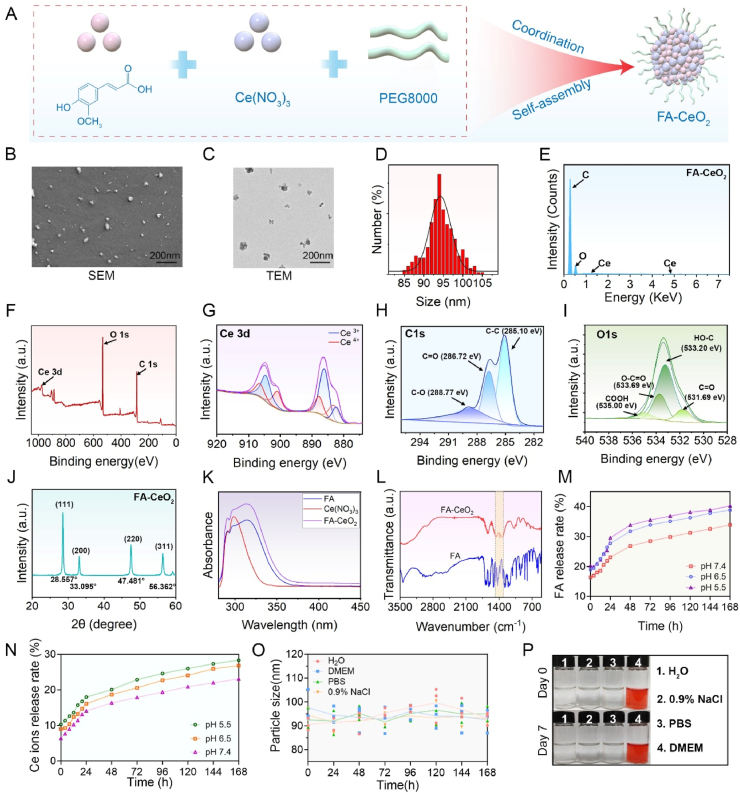
Synthesis and characterization of FA-CeO_2_. **a)** Schematic illustration of FA-CeO_2_ synthesis. **b,c)** Morphological characterization of FA-CeO_2_ via SEM (scale bar = 200 nm) **b)** and TEM (scale bar = 200 nm) **c)**. **d)** Hydrodynamic size distribution of FA-CeO_2_ assessed via DLS. **e)** Elemental composition mapping of Ce, C, and O in FA-CeO_2_. **f)** XPS patterns of FA-CeO_2_ with high-resolution spectra for Ce 3d **g)**, C 1s **h),** and O 1s **i)** peaks. **j)** XRD spectrum of FA-CeO_2_. **k)** UV–vis spectrum of FA-CeO_2_. **l)** FT-IR spectra comparing functional groups in FA and FA-CeO_2_. **m,n)** pH-dependent release values of FA **m)** and Ce ions **n)** from FA-CeO_2_ under physiological (7.4), mildly acidic (6.5), and acidic (5.5) conditions. **o)** Long-term colloidal stability of FA-CeO_2_ in various media, monitored via DLS over time (n = 3). **p)** Visual stability assessment of FA-CeO_2_ dispersions in water, PBS, 0.9 % NaCl, and DMEM at days 0 and 7.

The FA-CeO_2_ nanocomposites were made via a one-pot synthesis approach [20], integrating cerium nitrate hexahydrate (Ce(NO_3_)_3_·6H_2_O), ferulic acid (FA), and polyethylene glycol 8000 (PEG 8000) (Fig. 1A). Structural validation of FA-CeO_2_ was achieved through scanning electron microscopy (SEM) and high-resolution transmission electron microscopy (TEM), which illustrated spherical uniformly dispersed nanoparticles averaging 80 nm in size (Fig. 1B and C). Dynamic light scattering (DLS) analysis further confirmed stability, revealing a hydrodynamic diameter of 92 nm without aggregation (Fig. 1D). Elemental mapping confirmed the homogeneous distribution of carbon (C), oxygen (O), and cerium (Ce) (Fig. 1E).

X-ray photoelectron spectroscopy (XPS) identified Ce 3d, C 1s, and O 1s peaks (Fig. 1F–I), aligning with CeO_2_'s binding energies, thereby confirming successful nanocomposite formation. XPS further revealed an approximately 1:1 ratio of Ce^3+^/Ce^4+^ in CeO_2_ (Fig. 1G). The coexistence of Ce^3+^ and Ce^4+^ in FA-CeO_2_ is essential for their redox activity, with a higher ratio of Ce^4+^ improving catalase (CAT) activity and a higher ratio of Ce^3+^ enhancing superoxide dismutase (SOD) activity [[28], [29], [30]]. X-ray diffraction (XRD) patterns matched standard CeO_2_ references, with crystallographic planes (111), (200), (220), and (311) detected at 28.557°, 33.095°, 47.481°, and 56.362°, respectively [31], confirming retained crystallinity post-FA integration (Fig. 1J). Optical characterization via UV–Vis spectroscopy (Fig. 1K) highlighted FA's π-π∗ transition of the phenolic C=C bonds in the aromatic ring at 320 nm, and Ce(NO_3_)_3_ displayed significant absorption at 300 nm. FA-CeO_2_ showed two distinct absorptions at 320 nm, corresponding to the absorption of FA and CeO_2_'s absorption at 300 nm, demonstrating dual-component retention in FA-CeO_2_.

Fourier-transform infrared (FTIR) spectra revealed phenolic O–H stretching (∼3350 cm^−1^) and C=C vibrations (∼1600 cm^−1^), alongside hydroxyl bending (1300 cm^−1^) (Fig. 1L), suggesting stable FA-CeO_2_ coordination [20]. UV–Vis spectroscopic analysis of controlled release kinetics revealed a pH-dependent liberation profile for both Ce ions and FA. Under pH 5.5, sustained gradual release rates of 18 % increasing to 20 % for Ce ions and 30 % increasing to 34 % for FA were observed at 24 h and 48 h (Fig. 1M and N), demonstrating pH-responsive release behavior compatible with prolonged therapeutic delivery systems. Stability assessments in water, PBS, NaCl, and DMEM showed consistent particle size stability over 168 h, with no aggregation [23], further validated by 7-day room-temperature storage (Fig. 1O and P). The FA-CeO_2_ nanocomposites, synthesized through a streamlined one-pot method, demonstrated structural integrity, colloidal stability, and controlled release kinetics. These features spot FA-CeO_2_ as promising candidates for drug delivery systems, warranting further exploration of their therapeutic efficacy.

### 3.2 Antioxidant activity of FA-CeO_2_

**Fig. 2:**
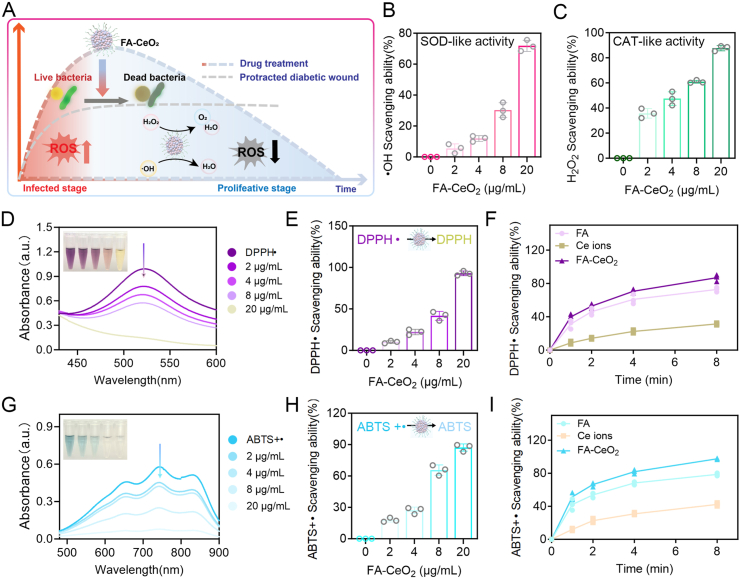
Antioxidant efficacy of FA-CeO_2_. **a)** Schematic illustration of FA-CeO_2_'s free radical scavenging mechanism. **b, c)** Dose-dependent scavenging efficiency of FA-CeO_2_ against •OH **b)** and H_2_O_2_**c)** (n = 3). **d)** UV–vis absorbance spectra of DPPH•·radicals following treatment with escalating concentrations of FA-CeO_2_. **e)** Quantification of DPPH• radical inhibition by FA-CeO_2_ across concentrations (n = 3). **f)** Time-dependent analysis of DPPH• radical scavenging by FA-CeO_2_ compared to free FA and Ce ions (n = 3). **g)** UV–vis absorbance spectra of ABTS^+^• radicals after exposure to increasing FA-CeO_2_ concentrations. **h)** ABTS^+^• radical scavenging efficiency at different concentrations of FA-CeO_2_ (n = 3). **i)** Time-dependent analysis of ABTS^+^• radical clearance capacity of FA-CeO_2_ compared to free FA and Ce ions (n = 3).

Nanozyme-based phytochemicals' antioxidant efficacy is crucial for scavenging active oxygen free radicals and mitigating lipid peroxidation at wound sites. Research has demonstrated that antioxidant nanomaterials significantly enhance wound repair by addressing oxidative stress [32]. Thus, the antioxidant efficacy of FA-CeO_2_ nanocomposites was assessed to measure their free-radical scavenging capacity toward hydroxyl radicals (•OH), hydrogen peroxide (H_2_O_2_), DPPH•, and ABTS^+^• radicals. Consistently, the results revealed that FA-CeO_2_ exhibited a progressive enhancement in scavenging •OH and H_2_O_2_ radicals as concentrations increased from 2 to 20 μg/mL (Fig. 2B and C), attributed to the redox-active ability of Ce^3+^/Ce^4+^ ions in CeO_2_, which catalyze ROS decomposition [33].

In the DPPH• assay, UV–vis absorption profiles demonstrated a dose-dependent reduction in radical intensity upon interaction with FA-CeO_2_ (Fig. 2D). Quantitative analysis further confirmed this result, with higher FA-CeO_2_ concentrations (20 μg/mL) yielding greater DPPH• inhibition (Fig. 2E). A time-dependent investigation revealed FA-CeO_2_'s rapid and sustained radical-scavenging ability (Fig. 2F) compared to free FA and Ce ions alone. The ABTS^+^• assay further confirmed FA-CeO_2_'s antioxidant potential. UV–vis spectra showed a concentration-dependent decline in absorbance, indicating efficient FA-CeO_2_'s ABTS^+^• scavenging (Fig. 2G). Quantitative results aligned with these findings (Fig. 2H), reinforcing FA-CeO_2_'s antioxidant activity. Time-dependent analysis underscored FA-CeO_2_'s rapid scavenging activity compared to FA or Ce ions alone (Fig. 2I). Antioxidant mechanisms typically involve distinct reactions where ABTS^+^• radicals are neutralized through electron transfer processes, while DPPH• radical scavenging primarily occurs via hydrogen atom transfer [34]. This suggests that FA-CeO_2_ effectively scavenges ABTS^+^• and DPPH•, indicating a synergistic mechanism involving electron transfer and hydrogen atom transfer (Fig. 2A).

FA-CeO_2_'s superior antioxidant performance stems from the synergistic interplay between CeO_2_ nanoparticles and FA. The Ce^3+^/Ce^4+^ redox cycling in CeO_2_ enables continuous electron transfer, effectively scavenging diverse ROS. Concurrently, FA enhances nanoparticle stability and dispersibility while contributing intrinsic antioxidant properties. This dual-action mechanism facilitates rapid ROS clearance and ensures prolonged activity, positioning FA-CeO_2_ as a promising therapeutic agent for mitigating oxidative stress and inflammation-related pathologies. These findings imply FA-CeO_2_'s potential in biomedical applications, particularly in contexts requiring strong antioxidant intervention. Its ability to efficiently scavenge multiple ROS types highlights its versatility and efficacy in addressing oxidative damage.

### 3.3 Antimicrobial activity of FA-CeO_2_

Diabetic wounds' susceptibility to bacterial infections is well-documented [35]. Research utilizing germ-free mice models has demonstrated accelerated wound epithelialization and angiogenesis alongside reduced scar formation, suggesting that mitigated pathogenic bacteria improve wound healing [36]. While nanozyme-based antimicrobial platforms have shown promise in diverse pathologies [37], the antibacterial potential of cerium-based nanozymes integrated with phytochemicals like FA in diabetic wounds remains underexplored. We thus ought to investigate the antibacterial efficacy of FA, Ce ions, and FA-CeO_2_ nanocomposites against *S. aureus* (Gram-positive) and *E. coli* (Gram-negative) using resazurin staining, bacterial colony counts, crystal violet biofilm quantification, live/dead fluorescence staining, and SEM.

**Fig. 3:**
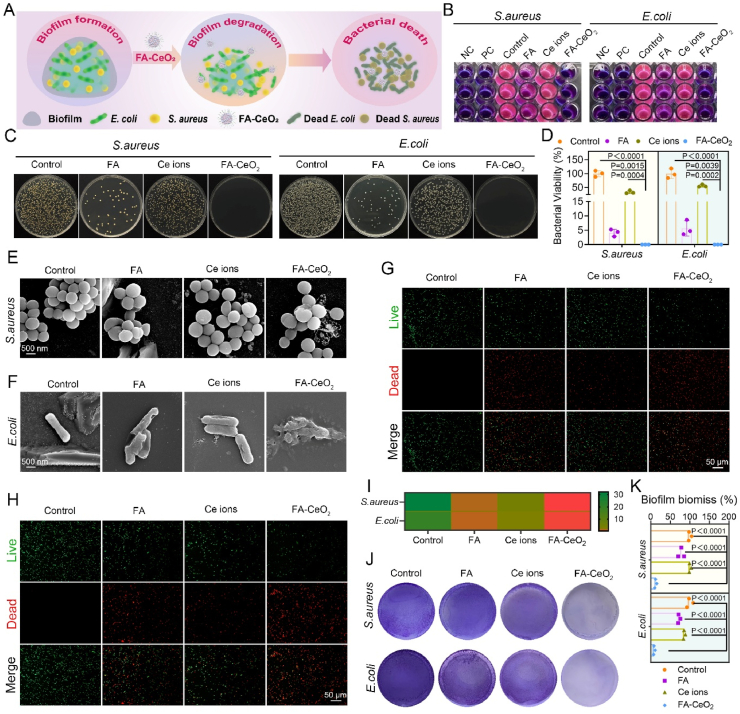
*In vitro* Antibacterial and antibiofilm activity of FA-CeO_2_. **a)** Schematic illustration of FA-CeO_2_'s antibacterial and antibiofilm effect. **b)** Metabolic activity of *S. aureus* and *E. coli* under different treatments, assessed via resazurin staining. **c, d)** Bacterial viability of the colony-forming unit (CFU) enumeration on agar plates **c)** for *S. aureus* and *E. coli* after treatment and quantitative analysis **d)**. **e, f)** SEM images illustrating morphological damage to *S. aureus***e),** and *E. coli***f)** after treatment with PBS, FA, Ce ions, or FA-CeO_2_. **g, h)** Live/dead fluorescence-based viability staining (live = green, dead = red) of *S. aureus***g)** and *E. coli***h). i)** Quantification of live/dead fluorescence ratios, highlighting FA-CeO_2_'s microbiocidal efficacy. **j, k)** Crystal violet staining of preformed biofilms **j)** and relative biomass quantification post-treatment **k)**. Data represent mean ± SD (n = 3), with different significance *p < 0.05, p < 0.01, and p < 0.001*.

Resazurin-based metabolic assays revealed significantly reduced fluorescence in FA-CeO_2_-treated groups (Fig. 3B), suggesting severe loss of bacterial viability. FA and Ce ions induced slight fluorescence reductions, whereas untreated controls retained significant metabolic activity. Agar plate counts revealed that FA-CeO_2_ nanocomposites achieved the highest antibacterial effect compared to FA and Ce ions treatment, suppressing 100 % of *S. aureus* and *E. coli* growth (Fig. 3C and D). These findings align with prior reports of enzyme-Ag-polymer nanocomposites (Ag/LBP) achieving a 97 % reduction in *S. aureus* and *E. coli* growth [38]. The metal-phenolic nanozyme CuTA demonstrated antibacterial rates of 93.13 % against *S. aureus* and 90.51 % against *E. coli* [37].

Furthermore, SEM imaging revealed smooth and intact cellular morphology in untreated *S. aureus* and *E. coli* groups, whereas FA-CeO_2_ treatment induced significant cell wall disruption and cellular structural deformation compared to FA or Ce ions treatment (Fig. 3E and F). This disruption is attributed to electrostatic interactions between cationic nanomaterials and anionic bacterial membrane components (e.g., teichoic acids in Gram-positive, lipopolysaccharides in Gram-negative species), which impede nutrient exchange and induce cytoplasmic leakage [39]. Similar mechanisms were observed in studies of PtCuTe nanosheets [3] and phospholipase-mimetic nanoceria (PAA-Cnp) [40]. BacLight™ live/dead assays confirmed FA-CeO_2_'s antibacterial efficacy, showing significant red fluorescence (dead cells) in FA-CeO_2_-treated groups. Conversely, most cells remained green (live cells) in controls and FA/Ce ions-treated groups (Fig. 3G–I). The turbidity of *S. aureus* and *E. coli* cultures incubated with FA, Ce ions, and FA-CeO_2_ (Fig. S1) supports previous findings, indicating that FA-CeO_2_ exhibits greater antibacterial activity than FA or Ce ions alone. This suggests a synergistic mechanism in which CeO_2_ nanoparticles disrupt bacterial membranes via oxidative stress, while FA may enhance this effect by further destabilizing membranes or interfering with metabolic pathways.

Given the importance of mouse wound models in understanding microbial biofilm function in wound healing [16,35], crystal violet staining was used to assess FA-CeO_2_'s antibiofilm activity. Results demonstrated a substantial decrease in biofilm formation for both pathogens after FA-CeO_2_ treatment (Fig. 3J and K), underscoring its dual inhibition action against bacterial growth and biofilm formation (Fig. 3A). Biofilm formation in wounds, a feature of bacterial infections, can affect fibroblast migration when the skin barrier is compromised [16]. Research has shown that *S. aureus* biofilms can impair fibroblast migration, keratinocyte function, and wound closure [36]. *S. aureus* is the most common bacterial infection in diabetic wounds, and *E. coli* impacts the depth of infection. Due to their biofilm formations, these strains are recognized as multidrug-resistant, complicating treatment [35]. In this study, FA-CeO_2_ demonstrated strong antibacterial activity against Gram-positive and Gram-negative bacteria. *S. aureus* has a thick peptidoglycan layer but lacks an outer membrane, potentially making it more susceptible to FA-CeO_2_'s oxidative damage. *E. coli* has a lipopolysaccharide-rich outer membrane, which may partially resist nanoparticle penetration. However, the strong inhibition observed implies that FA-CeO_2_ effectively overcomes this barrier.

These findings indicate that nanozyme-based phytochemicals like FA-CeO_2_ provide a transformative antimicrobial strategy thanks to their biomimetic scale, facilitating multivalent interactions with bacterial targets and offering a high surface area for cargo delivery. Their engineered structures can circumvent resistance mechanisms like efflux pumps and biofilm matrices. Collectively, FA-CeO_2_ exemplifies this potential by combining CeO_2_'s redox-modulating cerium ions (Ce^3+^/Ce^4+^ redox activity) with FA's highly phenolic hydroxyl groups to synergistically disrupt bacterial membranes and biofilms, suggesting that it could serve as a next-generation antibacterial agent for targeting bacterial infections.

### 3.4 ROS scavenging activities of FA-CeO_2_ in RAW 264.7 cells

**Fig. 4:**
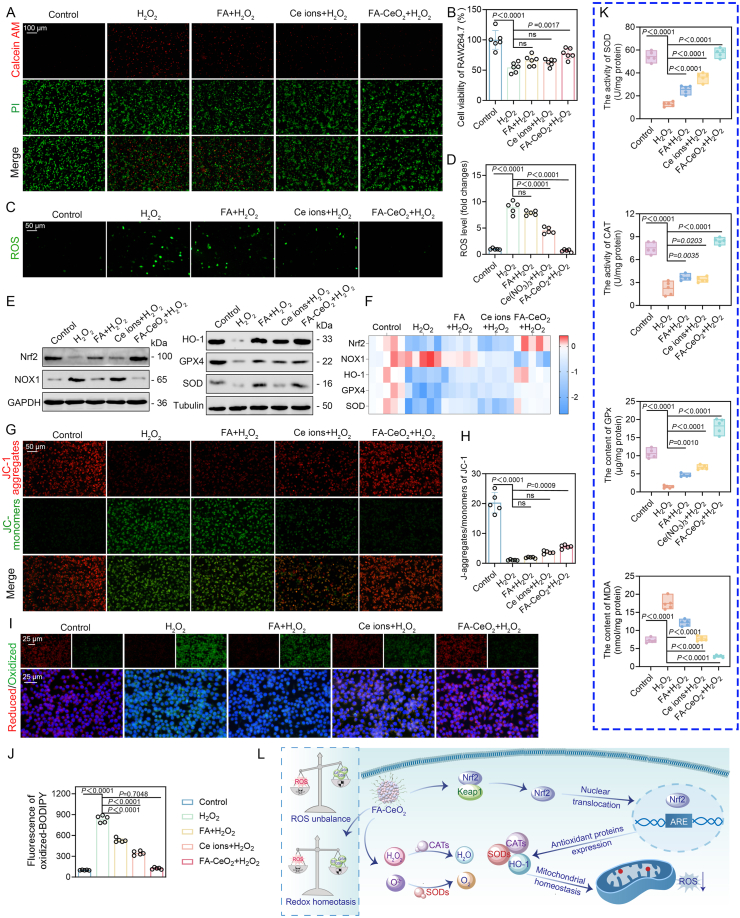
Redox regulation of FA-CeO_2_ in RAW 264.7 cells. **a, b)** Viability assessment via live/dead staining **a)** and quantitative cell viability analysis **b)** of cells pretreated with FA, Ce ions, or FA-CeO_2_ before H_2_O_2_ exposure (n = 6). **c, d)** ROS fluorescence images **c)** and quantitative analysis **d)** of RAW264.7 cells treated with FA, Ce ions, and FA-CeO_2_ followed by H_2_O_2_ exposure, respectively (n = 5). **e, f)** Western blotting **e)** and RT-qPCR analysis **f)** of antioxidants (Nrf2, HO-1, GPX4, and SOD) and ROS-generating (NOX1) markers levels under different treatments (n = 3). **g, h)** JC-1 staining to assess mitochondrial membrane potential (ΔΨm) **g)** and corresponding fluorescence intensity ratios (red/green) across treatment groups **h)** (Scale bar: 50 μm). **i,j)** Lipid peroxidation visualized via C11-BODIPY^581/589^-staining **i)** after different treatments and quantitative analysis of oxidative membrane damage **j)**. **k)** Antioxidant enzyme activity (SOD, CAT, GPx) and lipid peroxidation marker (MDA) levels in treated cells (n = 5). Ce ions and FA concentrations matched those in FA-CeO_2_ nanocomposites to ensure parity. **l)** Schematic illustration of the FA-CeO_2_ mechanism mediated the regulation of oxidative stress and antioxidant response via activation of the Nrf2 pathway in redox homeostasis. Data represent mean ± SD, with different significance *p < 0.05, p < 0.01, and p < 0.001*.

Redox homeostasis is critical in various human pathologies, primarily mediated through two distinct mechanisms [41]. ROS excessive generation directly disrupts cellular integrity by damaging nucleic acids, membrane lipids, structural proteins, and enzymatic functions, ultimately causing cellular or tissue dysfunction [42,43]. Conversely, dysregulated redox signaling in diabetic chronic wounds indirectly impedes healing by altering signal transduction pathways. In such contexts, H_2_O_2_ generated under pathological stimuli functions as a secondary messenger intricately tied to cellular redox [44]. To assess the potential of FA-CeO_2_ in redox regulation, RAW264.7 cells were subjected to H_2_O_2_-induced oxidative stress. CCK-8 assays revealed significant viability loss in H_2_O_2_-treated cells, while attenuated partial viability was observed in FA or Ce ions-treated groups. FA-CeO_2_ pre-treatment restored viability to near-normal levels (Fig. 4B), confirmed by live/dead staining showing a significant decrease in cell death (Fig. 4A).

Given the critical role of ROS in impairing diabetic chronic wound healing [45], we further quantified ROS levels using ROS-specific staining. As expected, ROS levels likely increased in H_2_O_2_-treated cells compared to controls, FA, and Ce ion treatments. (Fig. 4C and D). ROS levels increased in H_2_O_2_-treated cells but were significantly reduced by FA-CeO_2_, demonstrating its superior antioxidant properties. Concurrently, ROS-generating NOX1 protein levels were reduced, while the protein levels of antioxidant enzymes like HO-1, GPX4, and SOD increased (Fig. 4E and Fig. S2). WB analysis further revealed that FA-CeO_2_ treatment upregulated Nrf2 protein levels, a key antioxidant regulator [46]. RT-qPCR results were consistent with WB, showing increased Nrf2 mRNA expression in FA-CeO_2_-treated groups (Fig. 4F). Nrf2 also plays a key role in the onset and progression of several diseases [[47], [48], [49], [50]]. Nrf2 coordinates defenses against oxidative damage and promotes resilience to oxidative stress, activating the antioxidant response element (ARE) to upregulate genes such as HO-1, SOD, and CAT [46]. Sustained Nrf2 activation promotes wound re-epithelialization, with its effectiveness closely tied to the activation intensity. Moreover, the decrease in NOX1 levels and increase of antioxidant enzymes (HO-1, GPX4, and SOD) likely counteract oxidative stress, suggesting a moderate level of epithelial ROS acts as a sensor for Nrf2 expression. Consistent with this evidence, it suggests integrating ROS-scavenging ceria nanozymes with FA exhibits multi-enzymatic capabilities, alleviating oxidative damage effects to accelerate wound healing.

Considering that mitochondria are crucial organelles in generating ROS [51], we further assessed the mitochondrial membrane potential (ΔΨm) using JC-1 staining. The results substantially showed a loss of membrane potential in H_2_O_2_-treated cells. In contrast, those treated with FA-CeO_2_ effectively retained their potential (Fig. 4G and H), indicating a protective effect on mitochondrial function, consistent with previous research [33]. Lipid peroxidation, a non-reversible biochemical event indicative of necrotic cell death, typically manifests as a terminal consequence of oxidative stress-mediated injury [33]. The lipophilic fluorescent indicator C11-BODIPY^581/589^ was used as a ratiometric probe to investigate this process. Results revealed heightened oxidative damage in H_2_O_2_-treated cells, while FA-CeO_2_ significantly curtailed H_2_O_2_-triggered lipid peroxidation (Fig. 4I and J). Furthermore, FA-CeO_2_ restored SOD, CAT, and GPx activities while reducing MDA levels, countering H_2_O_2_-induced enzymatic depletion and lipid damage (Fig. 4K).

Catalase-mimetic nanozymes, such as those in FA-CeO_2_ nanocomposites, are recognized for their ability to catalyze the breakdown of H_2_O_2_ into O_2_ and H_2_O, thereby preventing peroxide accumulation and safeguarding tissues from oxidative damage [33]. Furthermore, SOD enzymes play a pivotal role in scavenging superoxide radicals (O_2_^−^•), catalyzing the dismutation reaction into H_2_O_2_ and O_2_, a critical defense mechanism against ROS in metabolic processes [33,48]. The SOD-like activity of FA-CeO_2_ highlights its therapeutic potential in managing oxidative stress-associated pathologies, offering a dual mechanism to stabilize redox homeostasis (Fig. 4L) and ameliorate cellular injury [42]. The FA-CeO_2_ nanocomposites' antioxidative synergy arises from CeO_2_'s redox-cycling cerium ions (Ce^3+^/Ce^4+^), which scavenge ROS, combined with FA's radical-neutralizing phenolic compounds. This dual-action mechanism strengthens cellular defenses, safeguarding mitochondria and lipid membranes and confirming the strong antioxidant activity of FA-CeO_2_. These findings establish FA-CeO_2_ as a promising therapeutic agent for oxidative stress-associated pathologies, warranting further mechanistic and translational exploration.

### 3.5 Cell migration and proliferation of FA-CeO_2_ in HUVEC cells

**Fig. 5:**
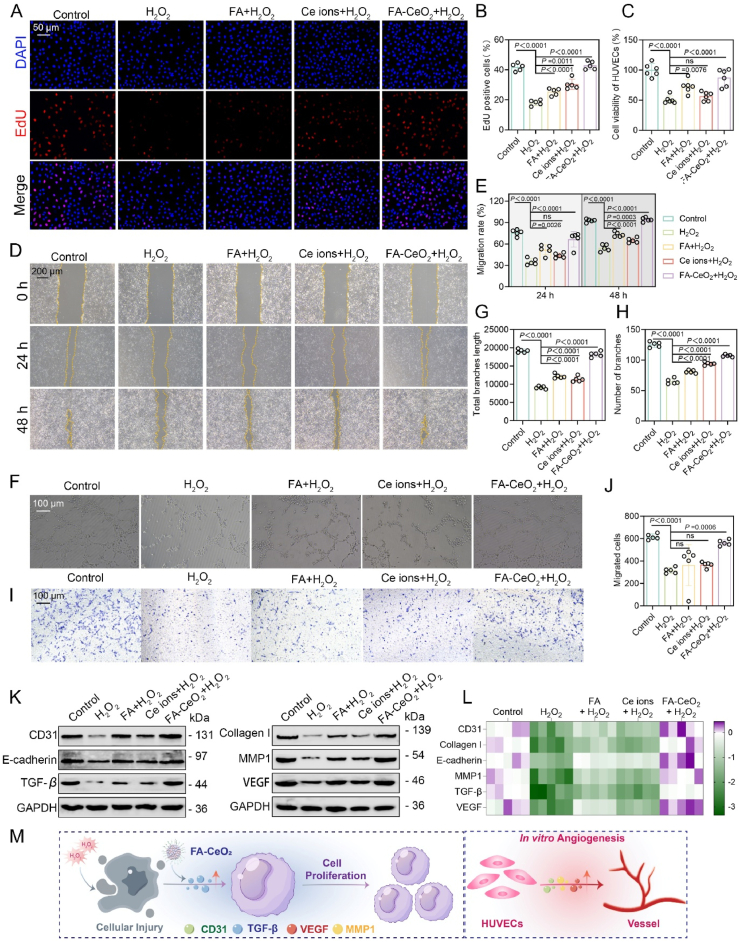
FA-CeO_2_ alleviates H_2_O_2_-induced endothelial cell dysfunction**. a, b)** Fluorescent images **a)** and quantitative analysis **b)** of EdU staining in HUVECs (Scale bar: 50 μm). **c)** Viability of HUVECs pretreated with FA, Ce ions, or CeO_2_-FA followed by H_2_O_2_ exposure (n = 5). **d, e)** Wound scratching assay **d)** and corresponding scratch closure rate quantification **e)** in HUVECs under different treatments (Scale bar: 200 μm). **f**-**h)** Tube formation assay **f)** and quantitative assessments of capillary-like structures **g, h)** (Scale bar: 100 μm). **i, j)** Representative migration images **i)** and quantitative analysis of migrated cells **j)** (Scale bar: 100 μm). **k, l)** Protein **k)** and mRNA expression levels **l)** of CD31, E-cadherin, TGF-β, Collagen I, MMP1, and VEGF. **m)** Schematic illustration of FA-CeO_2_ improved endothelial cell proliferation and *in vitro* angiogenesis post-cellular injury. Data represent mean ± SD, with different significance *p < 0.05, p < 0.01, and p < 0.001*.

Cell proliferation and migration are crucial for skin regeneration [52,53]. CeO_2_ nanoparticles have demonstrated significant potential in accelerating wound repair by enhancing cellular proliferation, migration, and ROS-scavenging in chronic ulcers [54]. To assess the cellular migration and proliferation potential of FA-CeO_2_ nanocomposites on wound healing, HUVECs were subjected to oxidative stress via H_2_O_2_ exposure. As expected, FA-CeO_2_ pre-treatment substantially enhanced cell proliferation, as evidenced by increased CCK-8 absorbance and EdU-positive cell counts (Fig. 5A–C), suggesting enhanced cell migration even under high oxidative stress, aligning with previous research [55]. Similarly, a cell scratch test further confirmed that FA-CeO_2_ treatment accelerated wound closure more effectively than FA or Ce ions treatments (Fig. 5D and E), while migratory capacity was compromised in H_2_O_2_-treated cells.

Angiogenesis is crucial for wound healing in diabetes, ensuring adequate nutrient and oxygen delivery to the wound site [21,56]. To investigate the pro-angiogenic effects of FA-CeO_2_, HUVECs were tested for migratory capacity using tube formation and transwell assay. Results showed that HUVECs exposed to H_2_O_2_ exhibited lower angiogenic potential, as evidenced by reduced capillary-like structure formation. FA-CeO_2_ treatment restored this capacity, leading to enhanced branch and length of tube formation, indicating strong endothelial cell function and vascularization support (Fig. 5F–H). Transwell migration analysis further aligns with the above results, revealing increased migratory potential in FA-CeO_2_-treated cells, with higher migrated cell counts compared to H_2_O_2_ groups. FA and Ce ions-treated groups showed less migration effectiveness than FA-CeO_2_ (Fig. 5I and J). These findings confirm that FA-CeO_2_ enhances cell migration and blood vessel formation *in vitro*, supporting its angiogenic role and improving oxygen supply in *vivo* wound healing [57].

Revascularization is an integral aspect of tissue regeneration, in which VEGF and CD31 stimulate the proliferation, expansion, and neovascularization of endothelial cells [58]. Western blotting and RT-qPCR analysis showed upregulation of angiogenesis-related proteins and mRNA expression of VEGF, CD31, E-cadherin, and extracellular matrix regulators (collagen I and MMP1) in FA-CeO_2_-treated cells (Fig. 5K–M and Fig. S3). Elevated TGF-β levels in FA-CeO_2_-treated cells further underscored pro-healing effects (Fig. 5K and L), consistent across transcriptional and translational levels. Collagen is a vital element in granulation tissue, with its synthesis by fibroblasts, regulated by transforming growth factor beta 1 (TGF-β1), being critical for efficient and accelerated wound repair [54]. E-cadherin, essential for endothelial cell adhesion and migration [59], was modulated by FA-CeO_2_, countering diabetic microvascular dysfunction. The enhanced wound healing and angiogenic properties of FA-CeO_2_ likely stem from the synergistic interplay of redox-active CeO_2_ nanoparticles, mitigating ROS and FA's capacity to scavenge free radicals, fostering a balanced cellular environment. This synergy activates critical pathways (VEGF, TGF-β, MMP1), fostering endothelial proliferation, migration, and vascular remodeling. Taken together, FA-CeO_2_ nanocomposites significantly enhance endothelial cell functions under oxidative stress, positioning them as promising therapeutic agents for wound healing and tissue regeneration, especially in oxidative injury contexts. Further exploration of signaling pathways could optimize clinical applications.

### 3.6 *In vivo* acute full-thickness cutaneous wound healing activity of FA-CeO_2_

**Fig. 6:**
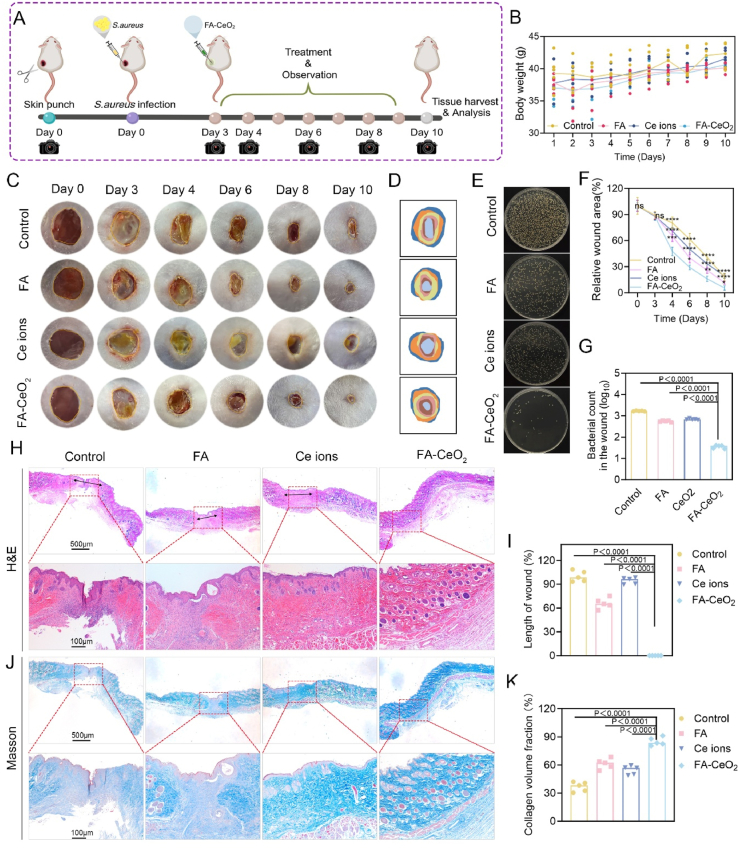
FA-CeO_2_ enhances the healing of acute full-thickness cutaneous wounds *in vivo*. **a)** Diagrammatic illustration of the experimental timeline for wound healing. **b)** Longitudinal monitoring of the mouse's body weight. **c, d)** Macroscopic images of wound healing, **c)** and wound closure progression illustration **d)**. **f)** Quantification of wound area reduction on days 0, 3, 4, 6, 8, and 10. **e, g)** Bacterial colony cultures, **e)** and CFU counts, **g)** from wound tissues across treatment groups at day 10. **h, i)** Histological analysis via H&E staining **h)** and scar length measurements **i)**. **j, k)** Collagen deposition was visualized by Masson's trichrome staining **j)** and corresponding quantitative collagen density **k)** (Scale bar: 100 μm). Data are expressed as mean ± SD (n = 5), with different significance *p < 0.05, p < 0.01, and p < 0.001*.

Wound repair encompasses various coordinated biological processes, including extracellular matrix synthesis, angiogenesis, re-epithelialization, contraction, and tissue remodeling. Disruptions in these mechanisms can prolong tissue damage and impede recovery [60]. To explore novel therapeutic strategies, we investigated the efficacy of FA-CeO_2_ nanocomposites in acute full-thickness cutaneous wound repair using a murine model with extensive infected wounds. As shown in Fig. 6A, treatments were immediately initiated post-wound induction (A full-thickness wound model of 8 mm) on day 0. Body weight remained stable across treatment groups, suggesting minimal systemic toxicity. Control mice exhibited slight weight loss, likely due to infection, whereas FA-CeO_2_ mice showed optimal weight maintenance, indicating enhanced recovery. Wound closure dynamics showed that FA-CeO_2_-treated wounds achieved near-complete closure by day 10, which is higher than the partial healing observed in the control and Ce ions groups and moderate progress in the FA group **(**Fig. 6C and D**)**. Quantitative wound area analysis further confirmed FA-CeO_2_ 's superior efficacy in accelerating healing, dropping to approximately 10 % of wound area by day 10 (Fig. 6F), consistent with previous research on nanozyme-enhanced healing [35,55].

Microbial load assessment was performed to strengthen the investigation. Results showed that FA-CeO_2_ significantly reduced bacterial counts compared to other groups, underscoring dual reparative and antimicrobial roles consistent with the *in vitro* results. Control wounds had the highest bacterial burden, correlating with delayed healing, while FA and Ce ions showed intermediate effects **(**Fig. 6E–G**)**. H&E staining of the wound section revealed advanced epithelialization and tissue organization in the FA-CeO_2_-treated group, with shorter wound lengths (Fig. 6H and I). Masson's trichrome staining demonstrated increased collagen I fiber deposition in FA-CeO_2_-treated mice, indicative of strong matrix remodeling, contrasting with lower levels in control and Ce ions groups and moderate levels in FA (Fig. 6J and K). Increased collagen-I signifies mature, mechanically stronger healing tissue and a lower risk of reinjury [61]. These findings align with previous research showing that ceria nanozyme platforms significantly enhance collagen fiber density and promote skin regeneration [58]. The synergy between CeO_2_ nanoparticles and FA underpins the therapeutic effects. CeO_2_'s redox activity mitigates oxidative stress, enhancing cell proliferation, while FA's anti-inflammatory properties aid immune regulation and tissue repair. FA-CeO_2_ also modulates key healing processes—angiogenesis, inflammation, and extracellular matrix remodeling—while exhibiting antimicrobial properties. These findings indicate that the dual antioxidative and antimicrobial properties of FA-CeO_2_ position them as a promising therapeutic for acute full-thickness cutaneous wounds and tissue regeneration challenges, warranting further clinical investigation.

### 3.7 *In vivo* diabetic chronic wound healing activity of FA-CeO_2_

**Fig. 7:**
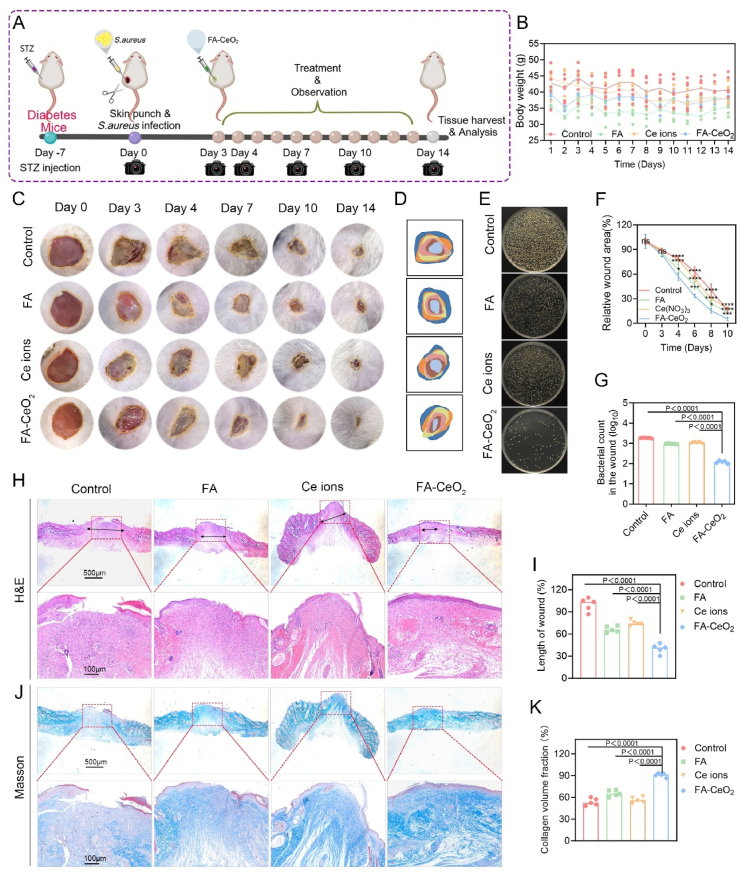
FA-CeO_2_ enhances the healing of diabetic chronic wounds *in vivo*. **a)** Diagrammatic illustration of the experimental timeline for diabetic wound healing. **b)** Longitudinal monitoring of the mice's body weight. **c, d)** Macroscopic images **c)** of wound healing and closure progression illustration **d)**. **f)** Quantification of wound closure on days 0, 3, 4, 7, 10, and 14. **e, g)** Bacterial colony cultures, **e)** and CFU counts, **g)** from wound tissues across treatment groups at day 14. **h, i)** Histological analysis via H&E staining **h)** and scar length measurements **i)**. **j, k)** Collagen deposition was visualized by Masson's trichrome staining **j)** and corresponding quantitative collagen density **k)** (Scale bar: 100 μm). Data are expressed as mean ± SD (n = 5), with different significance *p < 0.05, p < 0.01, and p < 0.001.*

Diabetic chronic wounds exhibit heightened susceptibility to microbial colonization, with hyperglycemia impairing fibroblast adhesion and delaying tissue repair [35]. To further strengthen the investigation, we assessed FA-CeO_2_ nanocomposites in a streptozotocin (STZ)-induced diabetic murine model. Mice with persistent hyperglycemia (>16.67 mmol/L for 7 days post-STZ) and standardized 8 mm chronic wounds (Fig. 7A) infected with *S. aureus* (1 × 10^8^ CFU/mL) were divided into four cohorts: control, FA, Ce ions, and FA-CeO_2_. Metrics such as body weight, wound closure rates, bacterial load, histological characteristics, and collagen deposition were assessed longitudinally. Treatments were initiated immediately post-wounding, with healing monitored over 14 days. All groups maintained consistent body mass, though controls exhibited a marginal reduction, likely due to infection-related stress. FA-CeO_2_-treated mice showed optimal mass retention, suggesting systemic biocompatibility and enhanced recovery (Fig. 7B).

Wound closure kinetics showed that FA-CeO_2_ significantly accelerated wound closure, achieving near-total epithelialization by day 14, outperforming partial healing in controls and Ce ions groups, and moderate progress in FA-treated mice (Fig. 7C, D, F). Quantitative wound area reduction confirmed FA-CeO_2_'s superior reparative capacity (Fig. 7F), dropping to approximately 7 % of wound area by day 14, consistent with previous research on nanomaterial-enhanced regenerative outcomes [45,62]. Furthermore, FA-CeO_2_ significantly suppressed bacterial proliferation compared to other groups, highlighting its dual antimicrobial and pro-healing roles. Controls exhibited the highest microbial burden, correlating with impaired reparative processes, while FA and Ce ions showed intermediate efficacy (Fig. 7E and G). To confirm the healing process, the wound section was processed on day 14 for investigation using H&E and Masson staining, as previously reported [45]. H&E staining revealed advanced re-epithelialization and tissue restoration in FA-CeO_2_-treated wounds (Fig. 7H), with significantly shorter wound lengths than controls and other groups (Fig. 7I). Moreover, Masson's trichrome staining demonstrated elevated collagen synthesis in FA-CeO_2_ mice (Fig. 7J), indicative of strong extracellular matrix framework formation. Quantitative analysis confirmed superior collagen deposition (Fig. 7K), which is critical for long-lasting tissue repair [63]. Such findings suggest that FA-CeO_2_ nanocomposites significantly enhance diabetic chronic wound repair through accelerated closure, microbial suppression, and optimized tissue remodeling. Collectively, FA-CeO_2_ nanocomposites show potential as a therapeutic candidate for diabetic wounds in place of traditional treatments.

### 3.8 Mechanism of FA-CeO_2_ mediates acute and chronic wound healing in diabetic mice

**Fig. 8:**
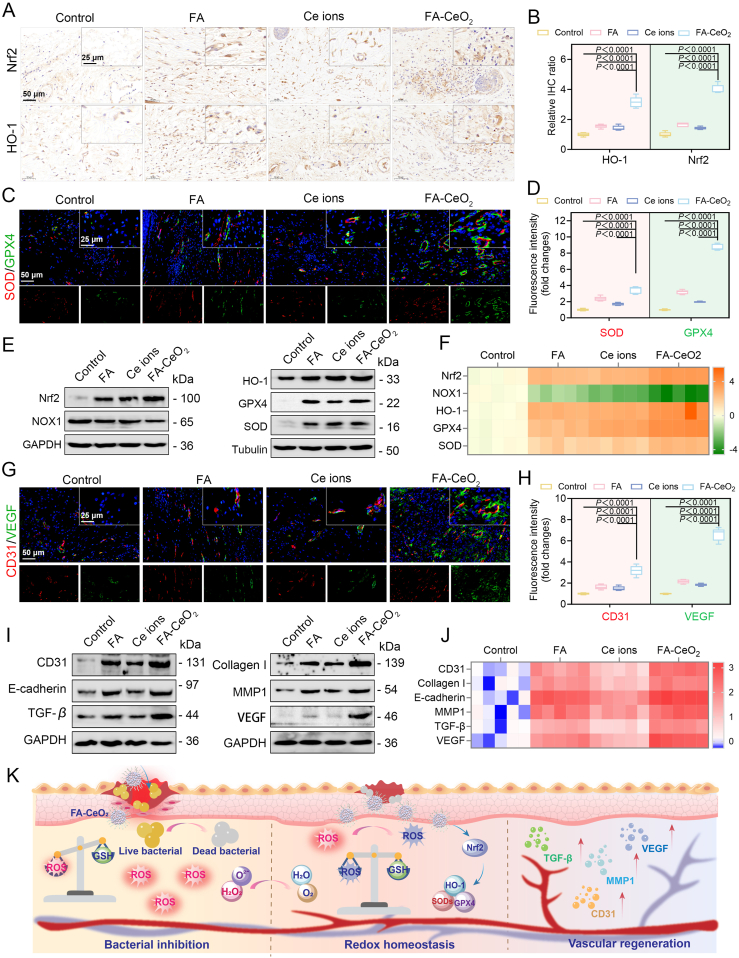
Mechanism of FA-CeO_2_-mediated acceleration of diabetic chronic wound healing. **a)** Immunohistochemical analysis (Inset depicts the corresponding high-magnification) and **b)** quantitative evaluation of Nrf2 and HO-1 expression in wound tissues (n = 3, Scale bar: 50 μm). **c)** Immunofluorescence staining (Inset depicts the corresponding high-magnification) and **d)** quantitative analysis of SOD and GPX4 expression levels (n = 3, Scale bar: 50 μm) **e)** Western blot analysis of Nrf2, NOX1, HO-1, GPX4, and SOD protein expression in FA-CeO_2_-treated diabetic wounds (n = 3). **f)** RT-qPCR quantification of Nrf2, NOX1, HO-1, GPX4, and SOD mRNA expression levels in diabetic wound tissue after FA-CeO_2_ treatment (n = 3). **g)** Immunofluorescence staining (Inset depicts the corresponding high-magnification) and **h)** quantitative analysis of CD31 and VEGF expression (n = 3, Scale bar: 50 μm). **i)** Western blot analysis of CD31, E-cadherin, TGF-β, Collagen I, MMP1, and VEGF protein expression in FA-CeO_2_-treated wounds (n = 3). **j)** RT-qPCR analysis of CD31, E-cadherin, TGF-β, Collagen I, MMP1, and VEGF mRNA expression levels (n = 3). **k)** Schematic representation of FA-CeO_2_-mediated acceleration of large-scale and diabetic chronic wound healing via antibacterial activity, redox regulation, and vascular regeneration. Data are expressed as mean ± SEM (n = 3), with different significance *p < 0.05, p < 0.01, and p < 0.001*.

Recent advancements in diabetic wound research highlight the critical role of molecular pathway modulation in enhancing chronic wound closure [35,55]. To unravel the mechanism basis of FA-CeO_2_ nanocomposites in diabetic wound repair, comprehensive acute and chronic wound infection models were created. The antioxidant nanozyme activity of FA-CeO_2_ was assessed through key regulators, including Nrf2, NOX1, HO-1, GPX4, and SOD. Antioxidant pathways are crucial in mitigating oxidative damage upon diabetic wound healing [64]. IHC staining revealed notably increased HO-1 and Nrf2 expression levels in FA-CeO_2_-treated wounds (Fig. 8A, Fig. S4), consistent with quantitative analysis (Fig. 8B, Fig. S5). This suggests activation of the cellular antioxidant system surrounding the wound tissue, as evidenced by similar research [64]. Dual IF staining further revealed enhanced GPX4 and SOD levels in FA-CeO_2_ groups (Fig. 8C, D, Figs. S6 and 7), aligning with evidence that NOX1 downregulation reduces ROS overproduction in diabetic wounds [64]. NADPH oxidases (NOXs) are a family of membrane enzymes primarily responsible for generating endogenous ROS and transferring electrons from NADPH to produce superoxide (O^2−^) [64]. Strong evidence indicates that ROS produced by NOX enzymes significantly contributes to oxidative damage in pathological conditions [64]. Thus, inhibiting these enzymes is a therapeutic strategy for treating various disorders.

Moreover, WB and RT-qPCR assays aligned with the above findings, showing increased Nrf2, HO-1, SOD, and GPX4 at both proteins (Fig. 8E, Figure S8A, and Figure S15**)** and mRNA levels (Fig. 8F, Figure S8B-F and Figure S9), underscoring FA-CeO_2_'s role in mitigating oxidative damage. The results further revealed that FA-CeO_2_ enhanced antioxidant enzyme activities of SOD, CAT, and GPx (Figure S10 and 17) while reducing the marker of MDA, highlighting its potential therapeutic role in conditions characterized by increased oxidative stress, such as diabetic wounds. GPX4 plays an essential role in neutralizing excess lipid peroxides, which are harmful byproducts of oxidative stress [64]. This evidence indicates that FA-CeO_2_ nanocomposites may impair oxidative damage and promote tissue repair through targeted modulation of the GPX4-associated signaling pathway. Accordingly, this study demonstrated FA-CeO_2_'s capacity to coordinately regulate the Nrf2/HO-1 antioxidant pathway and GPX4 activity. This dual regulatory effect reduces oxidative stress and enhances the wound healing process by fostering a regenerative microenvironment.

To further gain insight into the mechanism of cellular proliferation and migration, angiogenic potential was assessed via CD31 and VEGF dual IF staining (Fig. 8G). Results showed that FA-CeO_2_ significantly heightened CD31 and VEGF expression, as evidenced by quantitative analyses of IF (Fig. 8G and H, Figure S11, and Figure S12). WB further confirmed the upregulation of CD31, E-cadherin, TGF-β, collagen I, MMP1, and VEGF (Fig. 8I, Fig. S13A). Similarly, RT-qPCR aligned with WB results, showing upregulation of these biomarkers’ mRNA expression (Fig. 8J, Figure S13B-G, and Figure S14), indicating enhanced endothelial function and extracellular matrix (ECM) restructuring. The mechanism of wound healing progression (Fig. 8K) is orchestrated through dynamic interactions among various molecular entities, such as ECM, growth factors, and matrix metalloproteinases (MMPs). Critical aspects of tissue repair include cellular migration across the ECM and the MMP-mediated remodeling and breakdown of the ECM [65]. Collagen I, the predominant protein within the ECM, forms fibrous structures that confer mechanical strength to tissues. Conversely, MMPs, a family of zinc-dependent enzymes requiring calcium, play a central role in ECM degradation and are integral to all phases of wound healing [65]. During tissue remodeling processes such as wound repair, MMP expression is swiftly upregulated and transcriptionally activated by various cytokines and growth factors, including VEGF and TGF-β [65].

Moreover, this study demonstrates that the degradation of ECM during tissue healing accelerates the production of MMP-1 in keratinocytes at the wound's edge. Fibroblasts are the main source of MMP-1, an enzyme critical for breaking down collagen I [65], which promotes cell migration. Though common in chronic wounds, Elevated MMP-1 levels, as shown in this study, are vital for the healing process. Studies indicate that adding MMP-1 during repair can decrease scarring, favoring regenerative healing over simple wound closure [66]. As healing progresses, MMP-1 levels gradually decline, which is essential for proper tissue remodeling. This evidence suggests that FA-CeO_2_ nanocomposites promote wound closure and re-epithelialization by activating molecules like VEGF and TGF-β, potentially modulating MMP-mediated signaling pathways. Accordingly, FA-CeO_2_ facilitates diabetic wound healing by combining antioxidative effects through Nrf2/HO-1 activation with pro-angiogenic and extracellular matrix remodeling activities. It reduces oxidative stress, enhances vascularization via VEGF and CD31, and improves tissue integrity through TGF-β and collagen I modulation, making it a promising therapeutic approach for overcoming barriers in diabetic wound repair.

### 3.9 Biosafety and biochemistry evaluation of FA-CeO_2_

Biocompatibility is crucial for clinically translating nanomaterials in biomedical applications [67]. The biocompatibility and cytotoxicity potential of FA-CeO_2_ nanocomposites were thoroughly assessed using RAW264.7 cells and HUVECs. CCK-8 assays revealed no significant adverse effects on cell viability at concentrations up to 160 μg/mL (Fig. S18), demonstrating their biological compatibility at therapeutic doses. Live/dead staining of HUVECs further aligned with this finding, with a predominance of viable (green) cells and minimal detection of nonviable (red) cells (Fig. S19), implying excellent cytocompatibility of FA-CeO_2_ [37,55]. Similarly, CeO_2_-based nanomaterials exhibited low cytotoxicity in diverse cellular models, reinforcing their safety profile [33].

Hemolysis assays, a standard method for evaluating blood compatibility of biomaterials *in vitro*, revealed hemolysis rates below 5 % for FA-CeO_2_ across various concentrations (Fig. S20), consistent with prior research [32,37]. Even at 512 μg/mL, FA-CeO_2_ maintained excellent blood compatibility, showing negligible erythrocyte damage. As described in Fig. 6, Fig. 7B, longitudinal monitoring of murine body weight showed stability in FA-CeO_2_-treated groups compared to controls, FA, and Ce ions, suggesting no systemic toxicity or metabolic disruption. H&E staining further confirmed these findings, revealing no evidence of tissue or cellular damage in major organs such as the heart, liver, spleen, lungs, and kidneys in treated mice (Fig. S21), consistent with prior studies on nanozyme safety [32,68]. These findings collectively confirm the absence of acute or chronic toxicity in vital tissues, reinforcing the FA-CeO_2_ nanocomposites' suitability for nano-biomedical applications. Moreover, serum levels of aminotransferases, including aspartate aminotransferase (AST) and alanine aminotransferase (ALT), serve as critical biomarkers for detecting drug-induced liver injury [69]. To assess the potential systemic effects of FA-CeO_2_ nanocomposites, serum concentrations of liver and kidney function markers, such as ALT, AST, blood urea nitrogen (BUN), and creatinine (Crea), were measured and complemented by histopathological assessments conducted 24 h post-injection. As illustrated in Fig. S22, serum levels of these biomarkers remained within normal physiological ranges in FA-CeO_2_-treated mice, indicating no adverse effects on hepatic or renal function [69].

A comprehensive safety evaluation encompassing cellular toxicity assays, erythrocyte integrity assessments, histopathological examinations, and blood biochemistry profiles collectively confirmed the exceptional biocompatibility and low toxicity properties of FA-CeO_2_ nanocomposites. The absence of hemolytic activity (Fig. S20), organ-specific damage (Fig. S21), or cytotoxic effects at clinically relevant concentrations supports the potential clinical translation of FA-CeO_2_, particularly for applications in wound management and infection mitigation. These results position FA-CeO_2_ as a promising candidate for therapeutic application, emphasizing its safety and efficacy in biomedical contexts.

## 4 Conclusion

Innovative therapies that address oxidative stress and bacterial resistance in chronic diabetic wounds remain a critical priority. Phytochemical nanozymes integrating ferulic acid (FA) and cerium oxide (CeO_2_) present a promising dual-modality therapeutic strategy for managing acute full-thickness cutaneous and diabetic wounds by balancing antimicrobial efficacy with tissue regeneration. The FA-CeO_2_ nanozymes demonstrated structural stability, pH-responsive release, and potent antioxidant activity through Ce^3+^/Ce^4+^ cycling and FA-mediated radical scavenging, effectively reducing ROS levels while preserving antimicrobial efficacy. Enhanced antimicrobial activity against Gram-positive and Gram-negative pathogens and biofilm disruption addressed critical challenges in diabetic wounds. Mechanistically, the nanozymes activated the Nrf2/HO-1 pathway, leading to upregulated VEGF/CD31 expression, accelerated cell proliferation, and collagen remodeling, supporting tissue regeneration. Comprehensive biosafety assessments confirmed minimal cytotoxicity, hemocompatibility, and no systemic toxicity, underscoring their clinical translation potential. Future studies should focus on optimizing formulation stability, elucidating long-term regenerative mechanisms, and validating therapeutic efficacy in clinical models to fully harness the dual antibacterial and regenerative potential of phytochemical nanozymes in chronic wound management.

## CRediT authorship contribution statement

**Yipeng Pang:** Data curation, Investigation, Validation, Writing – original draft. **Fructueux Modeste Amona:** Validation, Writing – original draft. **Xiaohan Chen:** Data curation, Investigation, Validation. **Yuxin You:** Data curation, Investigation, Validation. **Ziqi Sha:** Data curation, Investigation, Validation. **Zilu Liu:** Data curation, Investigation, Validation. **Jiamin Li:** Data curation, Investigation, Validation. **Yi Liu:** Conceptualization, Supervision, Writing – review & editing. **Xingtang Fang:** Conceptualization, Supervision, Writing – review & editing. **Xi Chen:** Conceptualization, Funding acquisition, Supervision, Writing – review & editing.

## Ethics approval and consent to participate

The authors declare that all Animal experiments complied with the National Institutes of Health's Guide for the Care and Use of Laboratory Animals and were approved by the Animal Ethics Committee of Jiangsu Normal University (Assigned Protocol Number: JSNU-IACUC 2025026). All authors comply with all relevant ethical regulations.

## Data availability statement

The data that support the findings of this study are available in the Supporting Information of this article.

## Funding

This research was funded by the 10.13039/501100001809National Natural Science Foundation of China (No. 32000108), 10.13039/501100004608Natural Science Foundation of Jiangsu Province (No. BK 20201022), the Key Research and Development Plan (Modern Agriculture) Project of Xuzhou City (No. KC22076), and Natural Science Research of Jiangsu Higher Education Institutions of China (20KJB180006).

## Declaration of competing interest

The authors declare that they have no competing financial interests or personal relationships that could have appeared to influence the work reported in this paper.
