# Supplementary material for: Phytochemical nanozymes reprogram redox for balanced antimicrobial and regenerative therapy in acute and chronic diabetic wounds
Source: Redox Biol. 2025 Jun 6;85:103718. doi: 10.1016/j.redox.2025.103718 (PMC12205665; doi:10.1016/j.redox.2025.103718)
Supplement: Multimedia component 1 [file mmc1.docx]

**Supplementary figures**


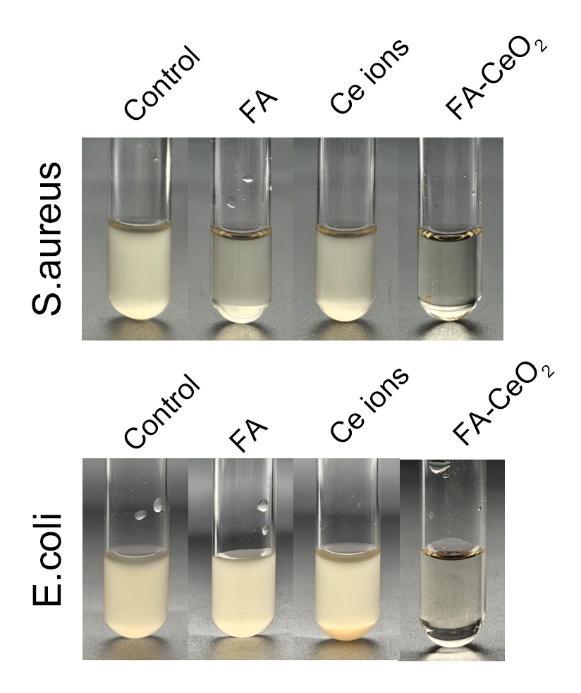


**Figure S1.** Antibacterial activity of FA, Ce ions, and FA-CeO_2_ against S. aureus and E. coli in centrifugal tube assays.


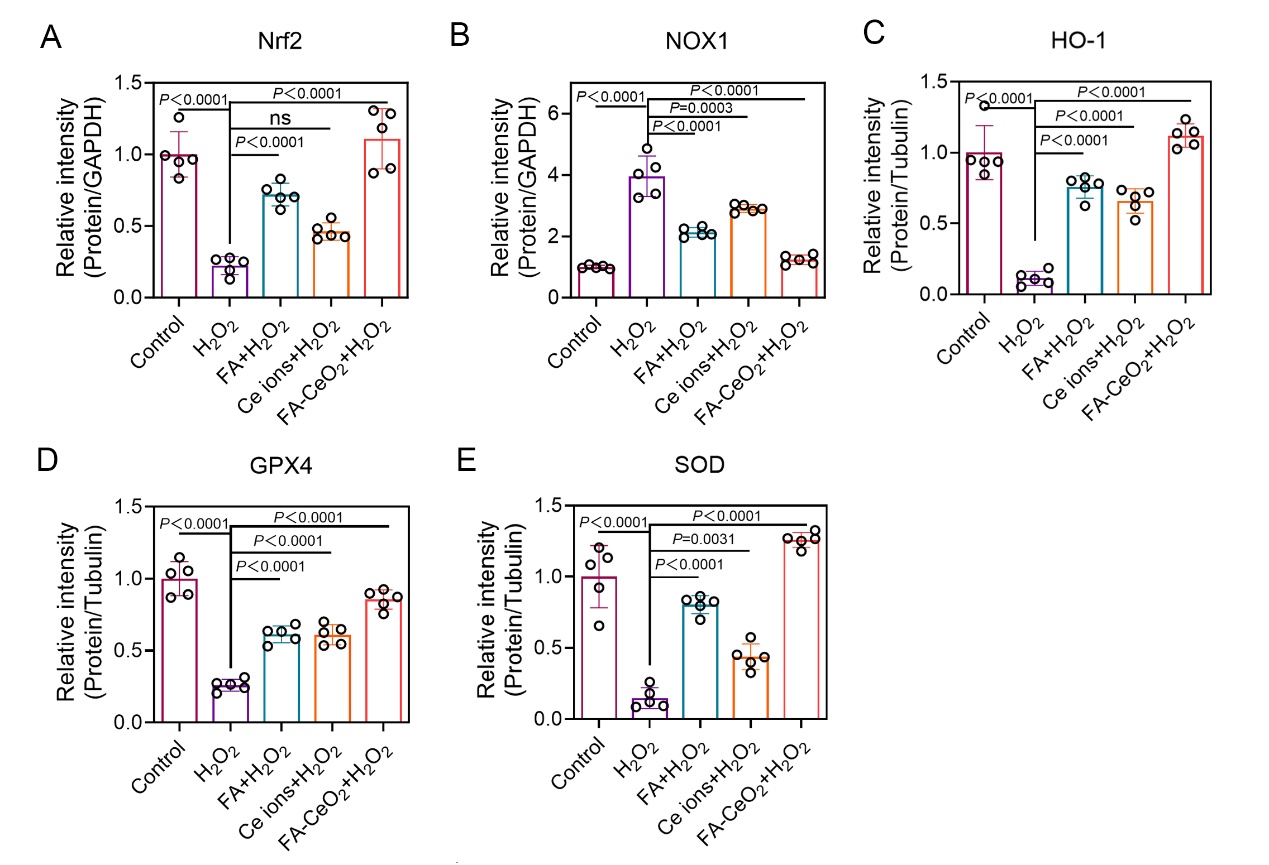


**Figure S2.** Quantitative intensity of Nrf2, NOX1, HO-1, GPX4, and SOD protein expression in RAW264.7 cells under different treatments (n = 5).


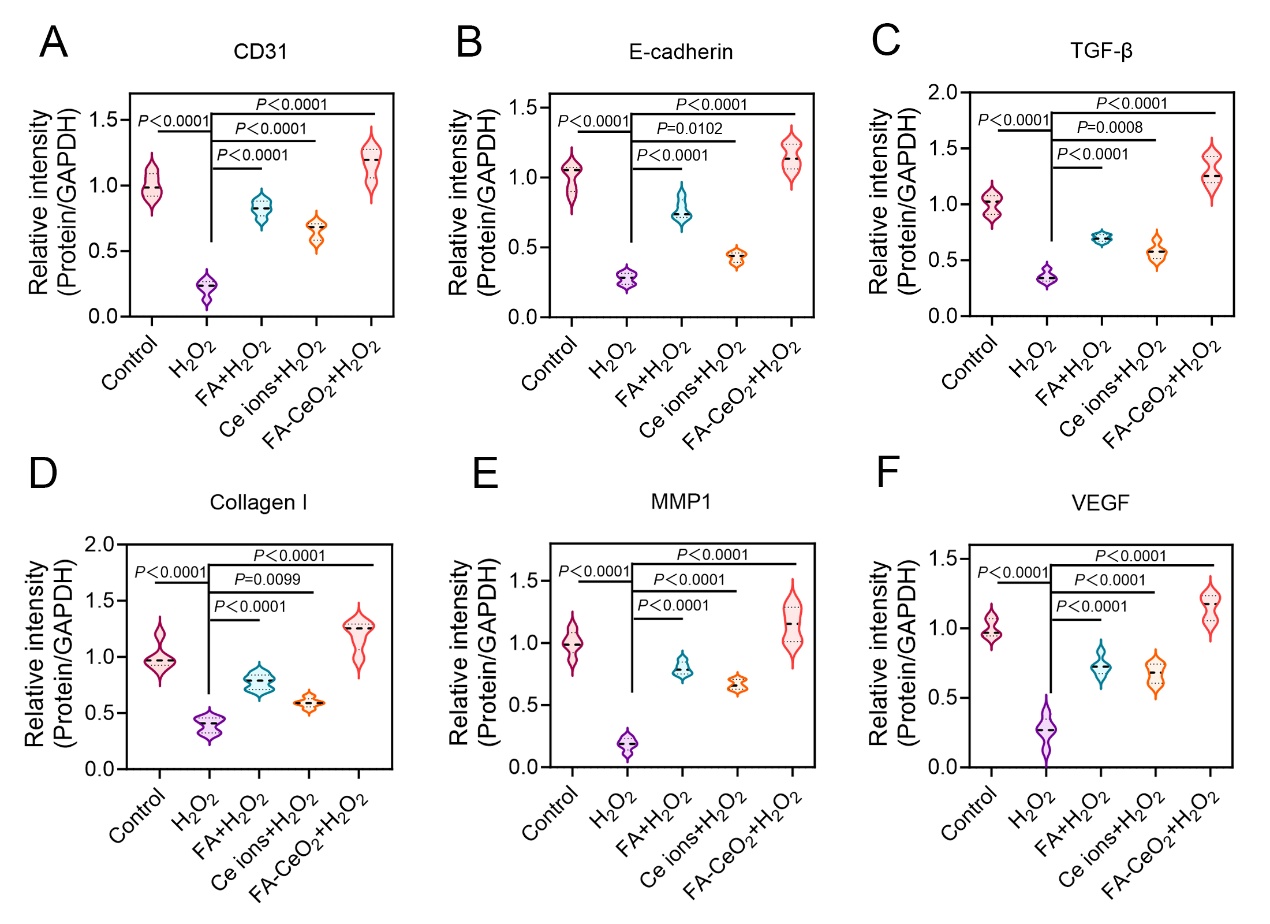


**Figure S3.** Quantitative intensity of CD31, E-cadherin, TGF-β, Collagen I, MMP1, and VEGF protein expression levels in HUVEC cells (n = 3).


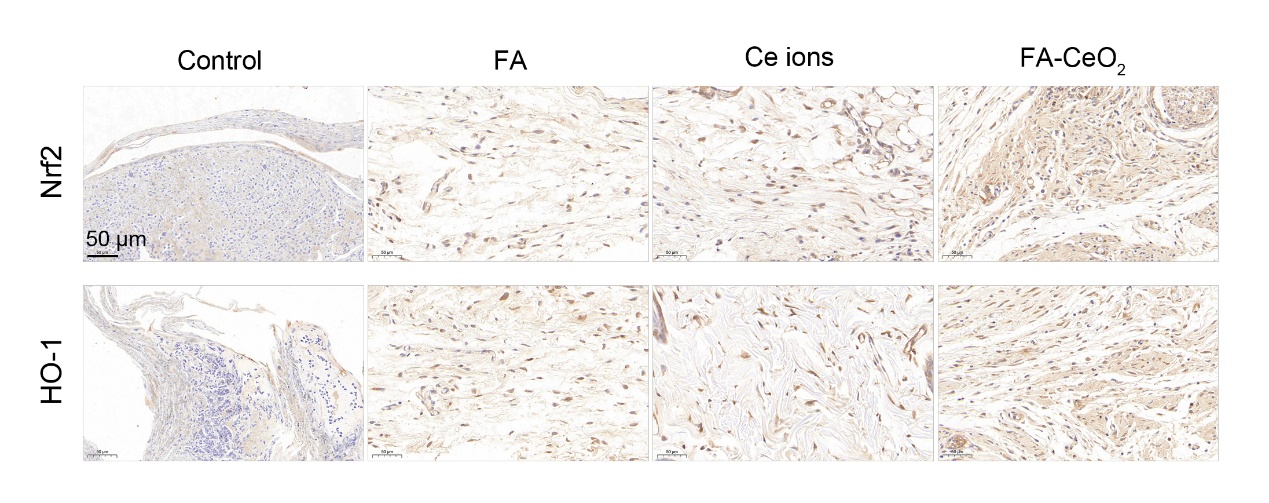


**Figure S4.** The molecular mechanism analysis of accelerating acute wound closure. Immunohistochemistry staining of Nrf2 and HO-1 expression in acute wound tissue (Inset depicts the corresponding high-magniﬁcation). n = 5, scale bar: 50 μm.


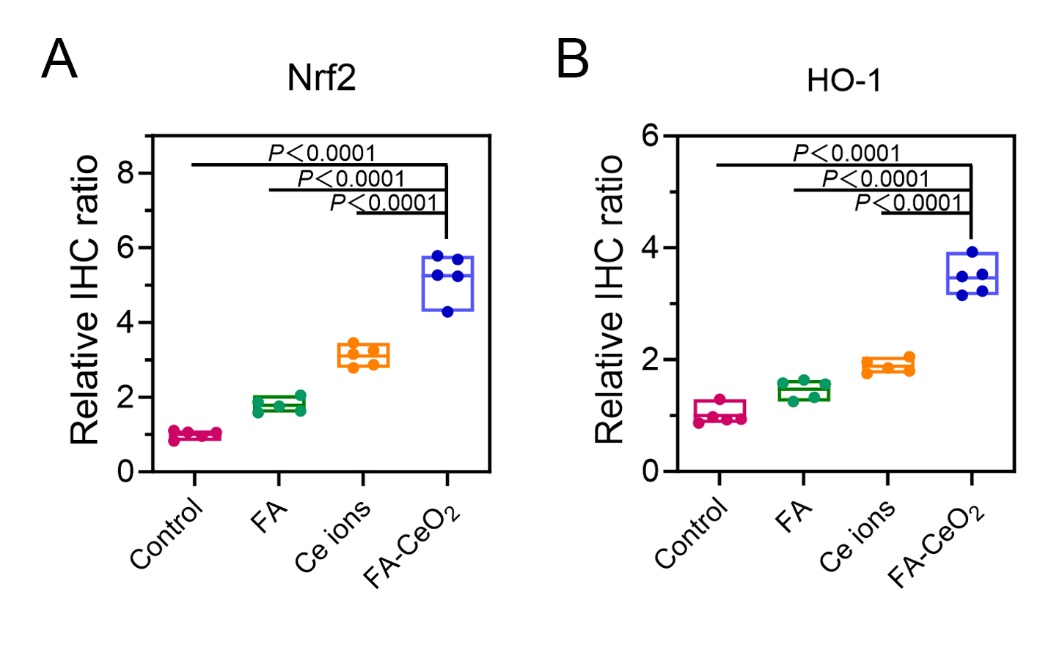


**Figure S5.** Quantitative analysis of Nrf2 and HO-1 expression in acute wound tissue using immunohistochemistry staining (n = 5).


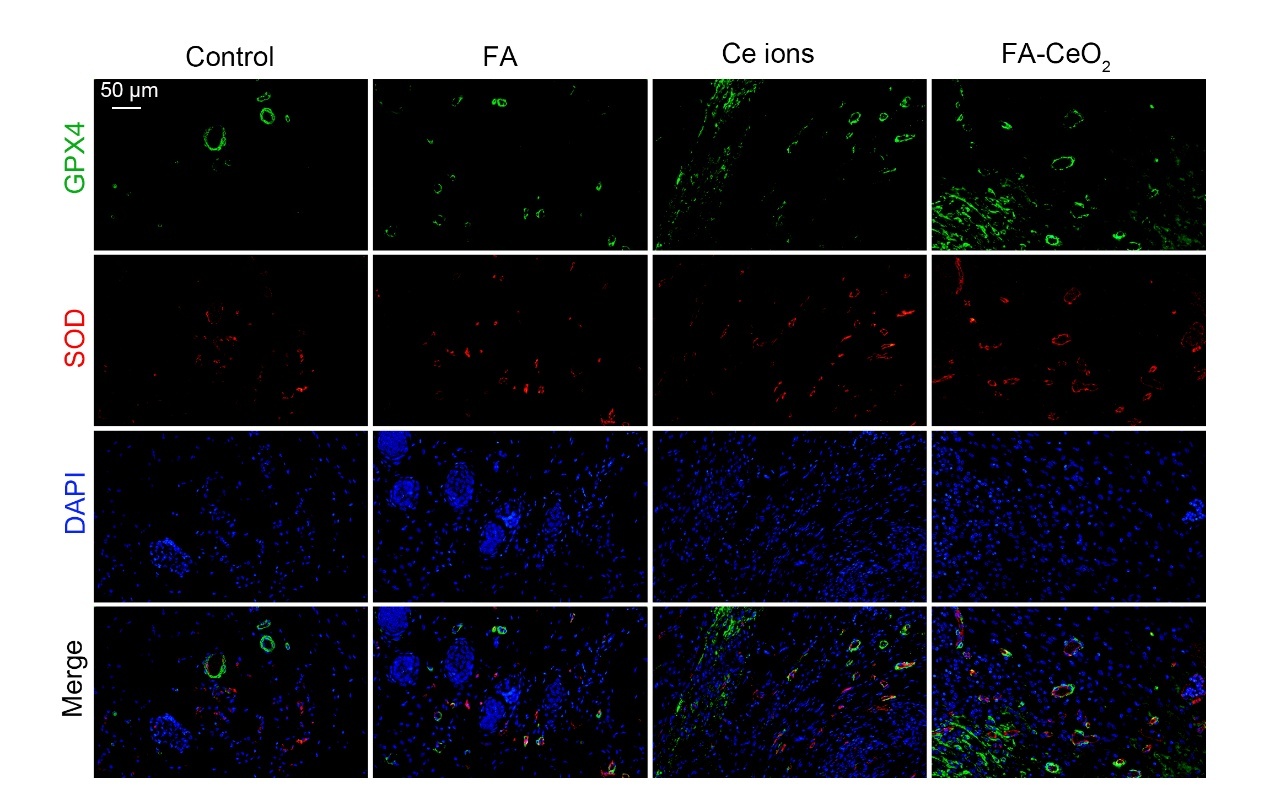


**Figure S6.** Immunofluorescence staining of SOD and GPX4 in acute wound tissue (Inset depicts the corresponding high-magniﬁcation). n= 5, scale bar: 50 μm.


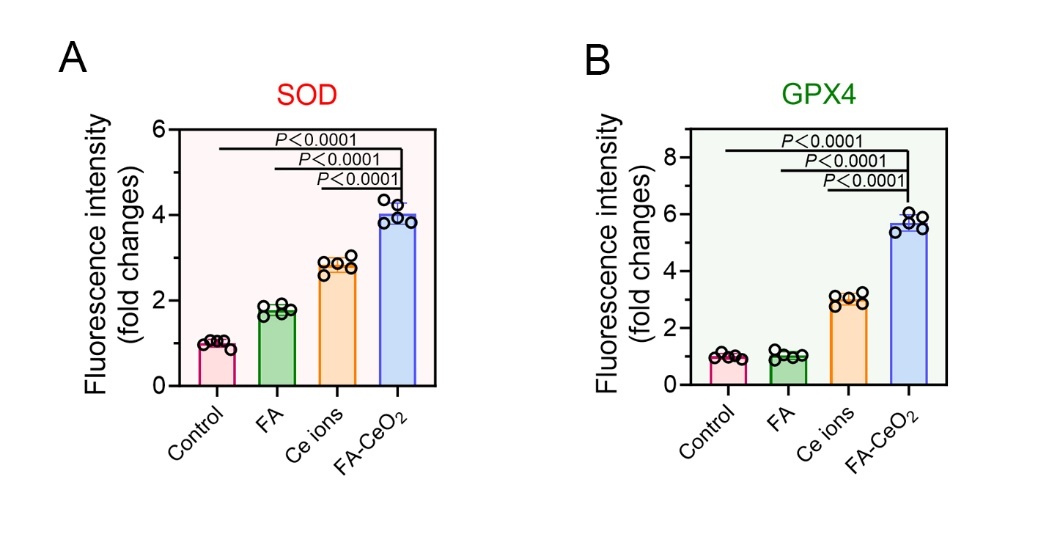


**Figure S7.** Quantification of SOD and GPX4 immunofluorescence intensity in acute wound tissue (n = 5).


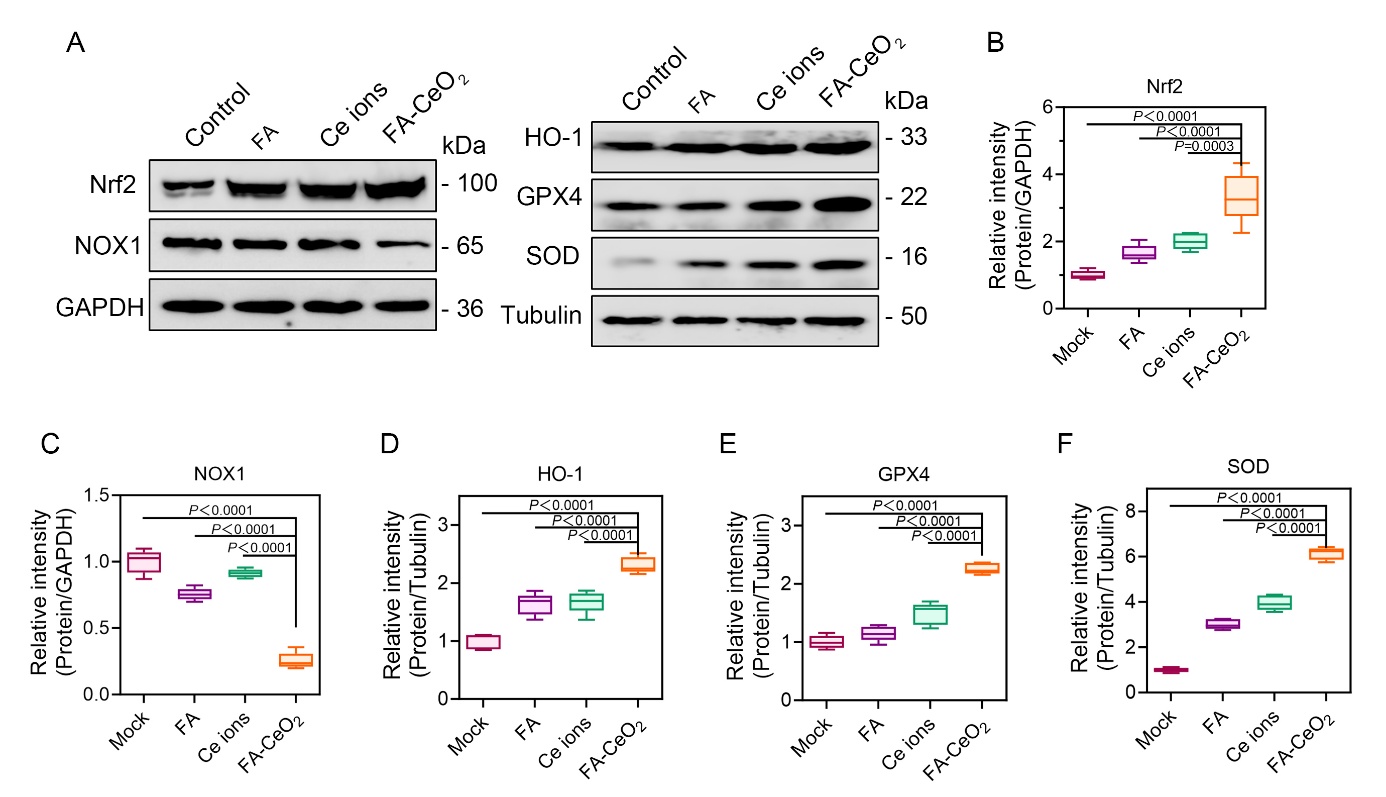


**Figure S8.** **(A)** Western blot and **(B–F)** quantitative analysis of Nrf2, NOX1, HO-1, GPX4, and SOD proteins expression levels in acute wound tissue after FA-CeO_2_ treatment (n = 5).


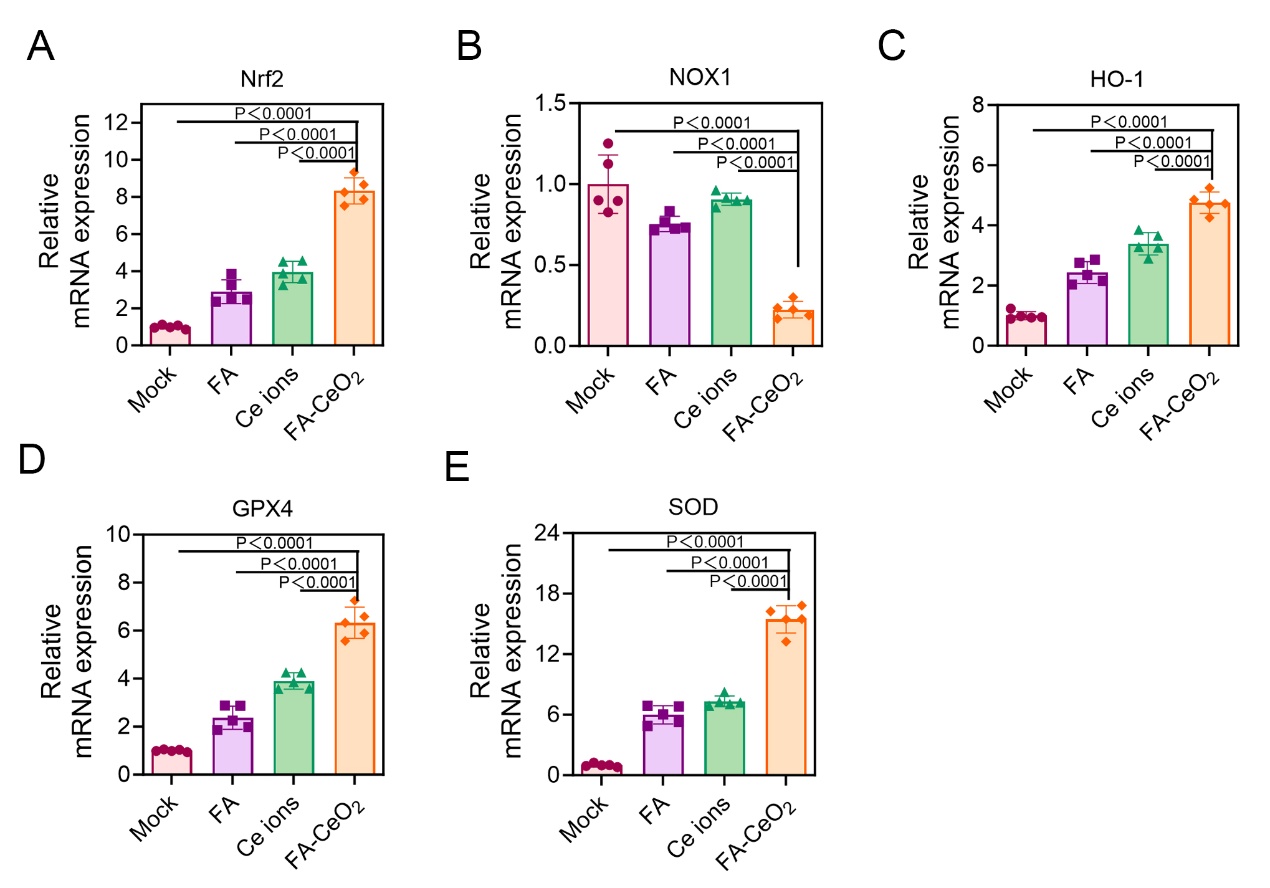


**Figure S9.** RT-qPCR quantification of Nrf2, NOX1, HO-1, GPX4, and SOD mRNA expression levels in acute wound tissue after FA-CeO_2_ treatment (n = 5).


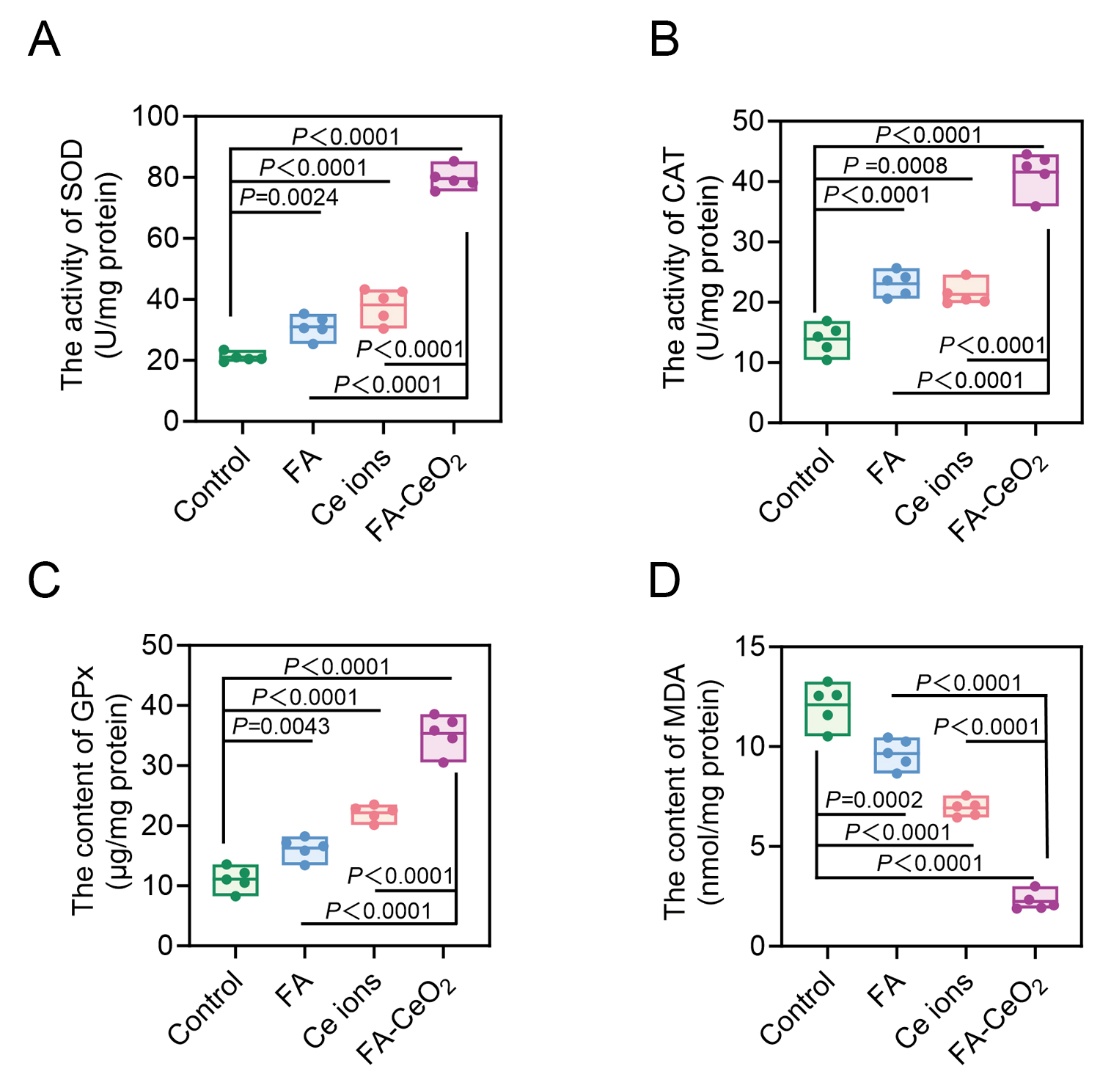


**Figure S10.** Enzymatic activity of SOD, CAT, GPx, and MDA expression levels in acute wound tissue after FA-CeO_2_ treatment, measured via assay kits (n = 5).


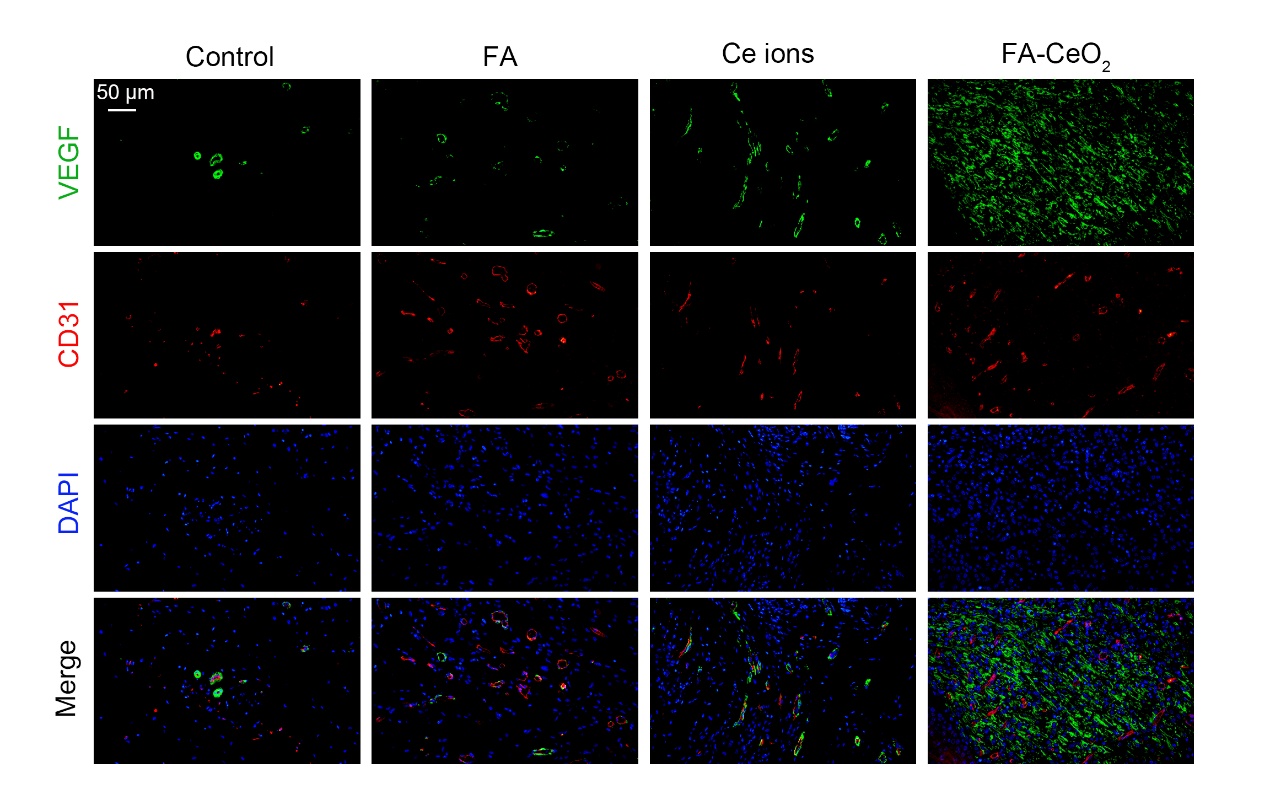


**Figure S11.** Immunofluorescence staining of CD31 and VEGF in acute wound tissue (Inset depicts the corresponding high-magniﬁcation). n= 5, scale bar: 50 μm.


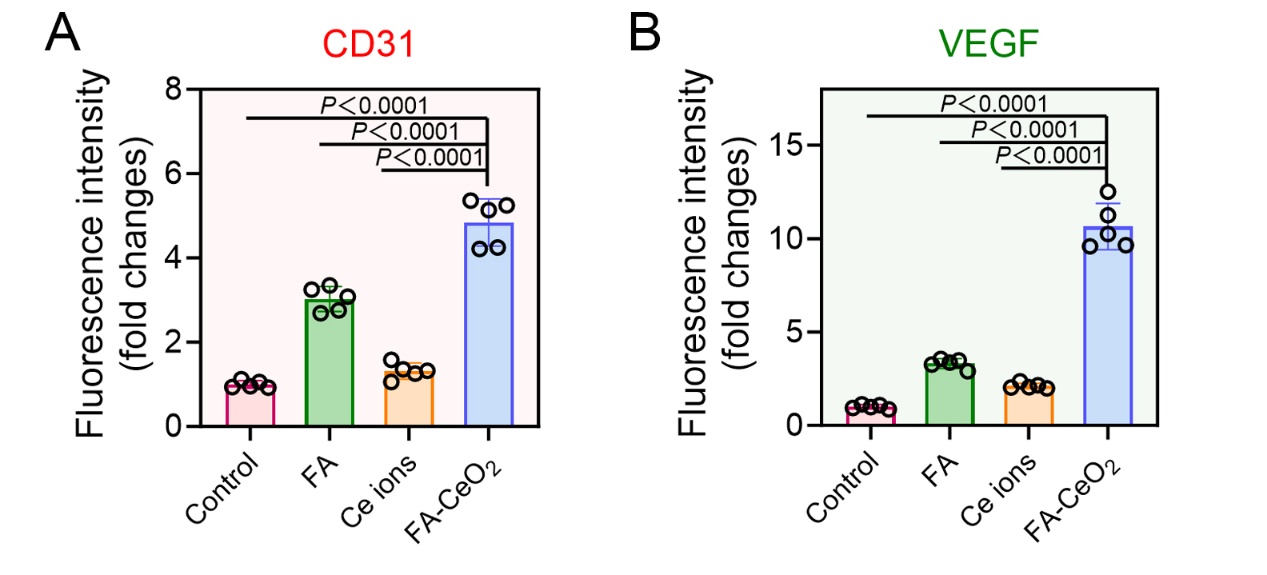


**Figure S12.** Quantitative analysis of CD31 and VEGF immunofluorescence intensity in acute wound tissue (n= 5). Scale bar: 50 μm.


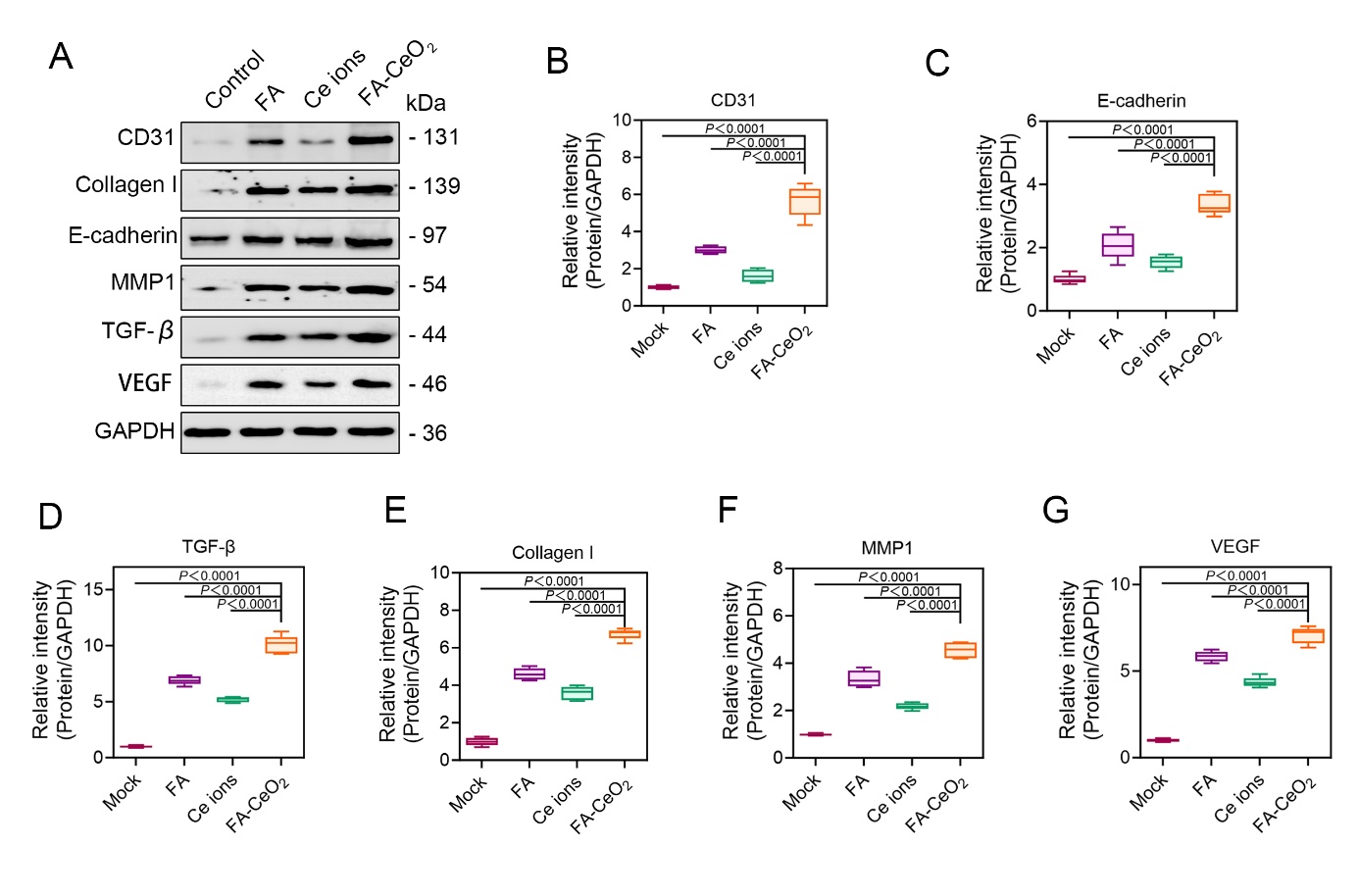


**Figure S13.** **(A)** Western blot and **(B–G)** quantitative analysis of CD31, E-cadherin, TGF-β, Collagen I, MMP1, and VEGF protein expression levels in acute wound tissue after FA-CeO_2_ treatment (n = 5).


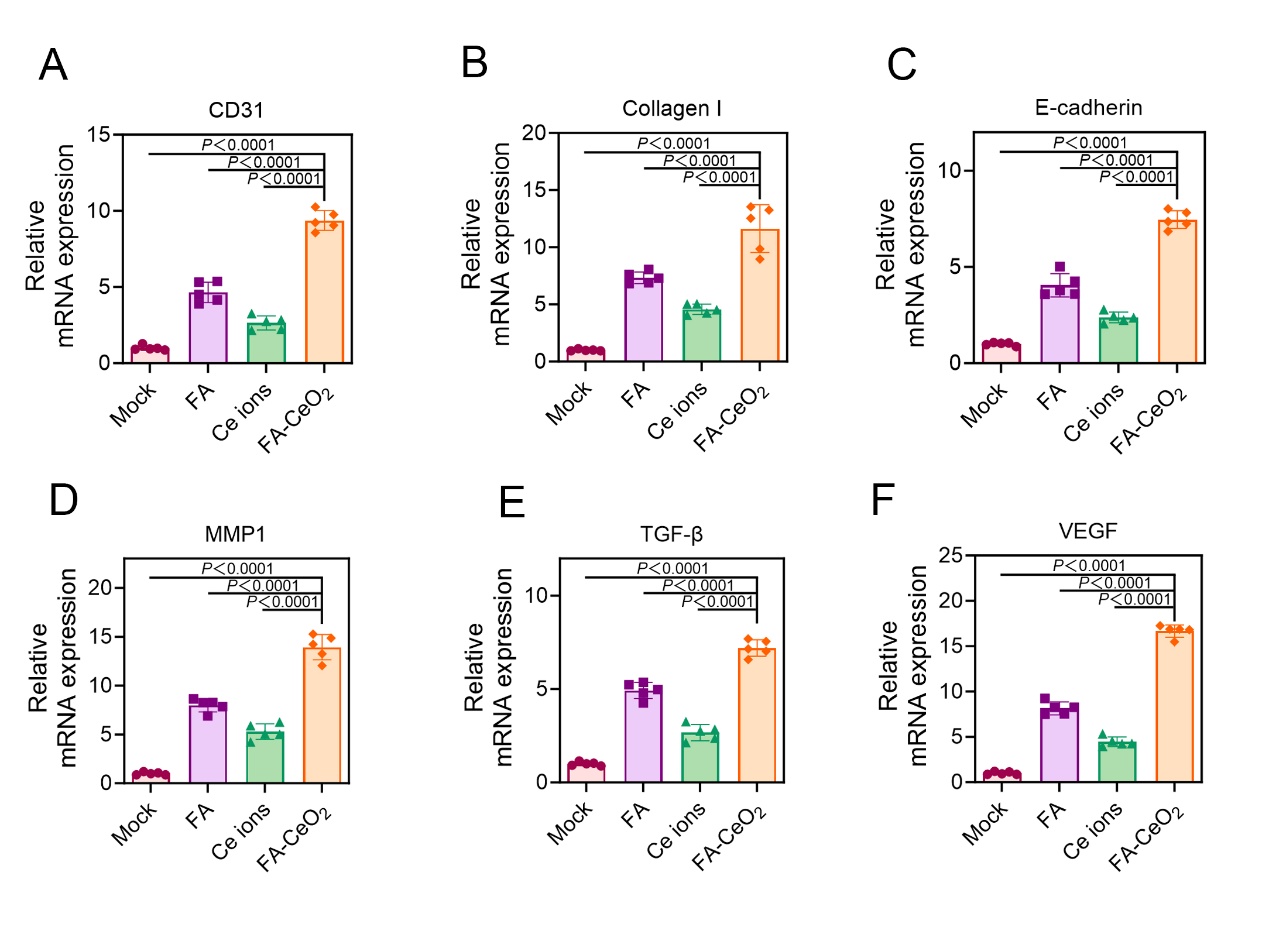


**Figure S14.** RT-qPCR analysis of CD31, Collagen I, E-cadherin, MMP1, TGF-β, and VEGF mRNA expression levels in acute wound tissue after FA-CeO_2_ treatment (n = 5).


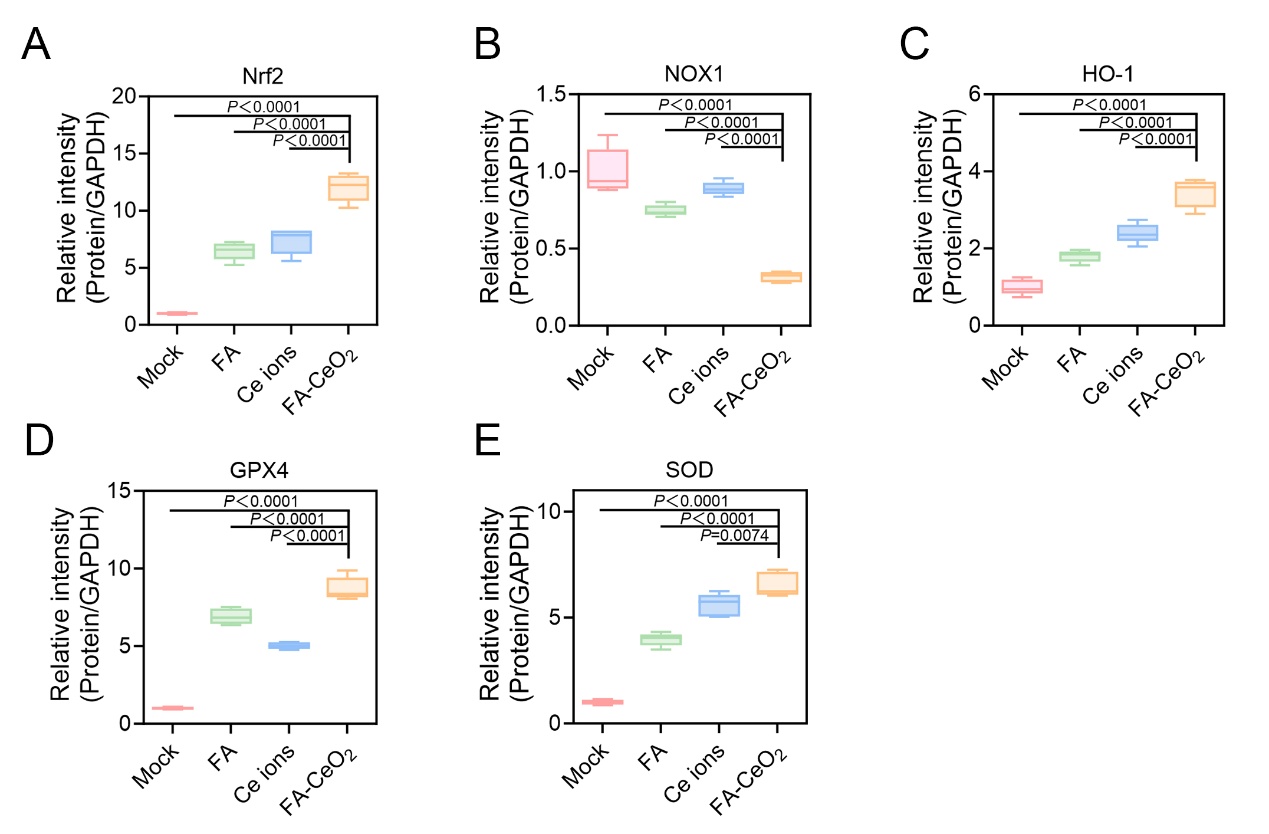


**Figure S15.** **(A-E)** Quantitative analysis of Nrf2, NOX1, HO-1, GPX4, and SOD proteins expression in diabetic chronic wound tissue after FA-CeO_2_ treatment (n = 5).


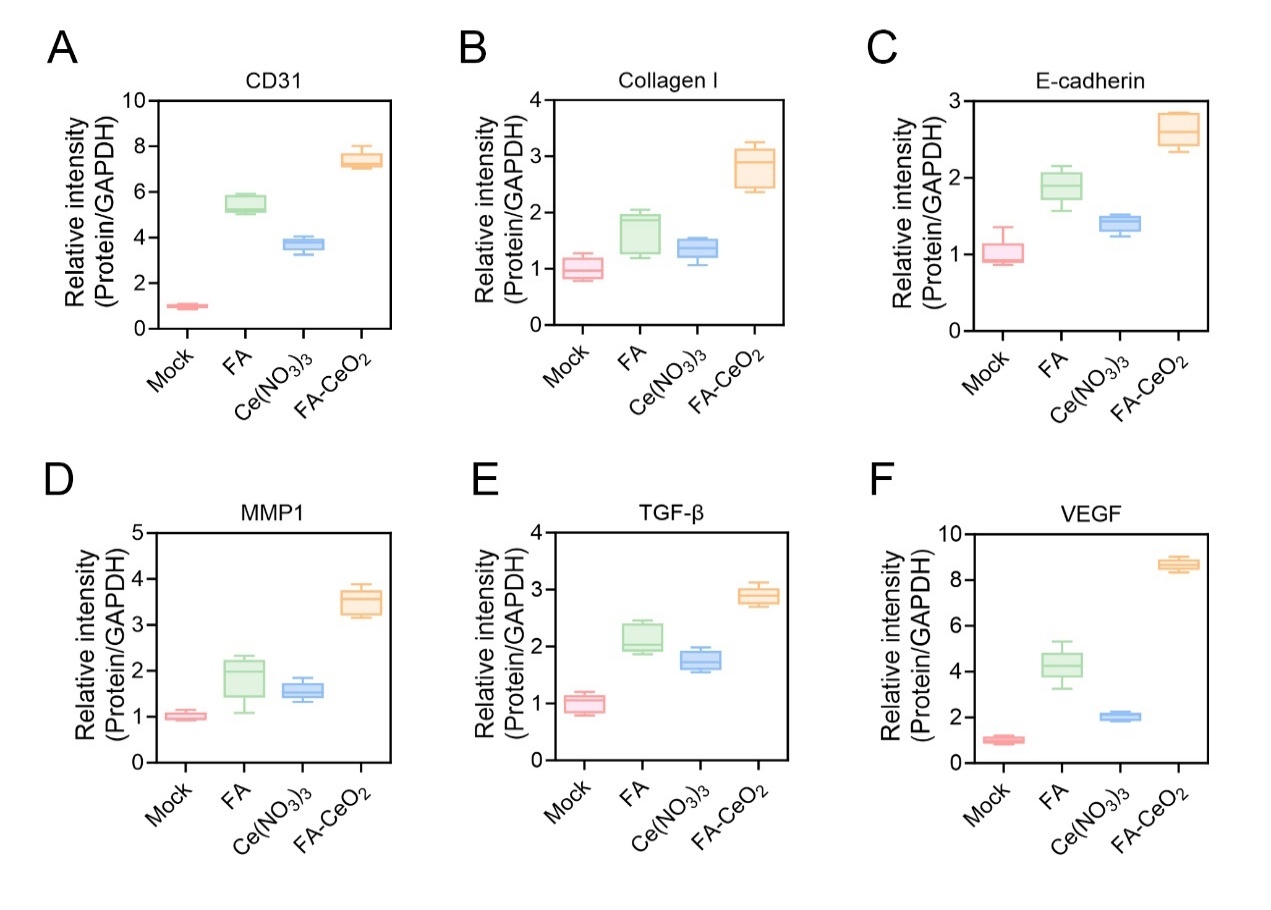


**Figure S16.** (A-F) Quantitative analysis of CD31, Collagen I, E-cadherin, MMP1, TGF-β, and VEGF protein expression in diabetic chronic wound tissue after FA-CeO_2_ treatment WB (n = 5).


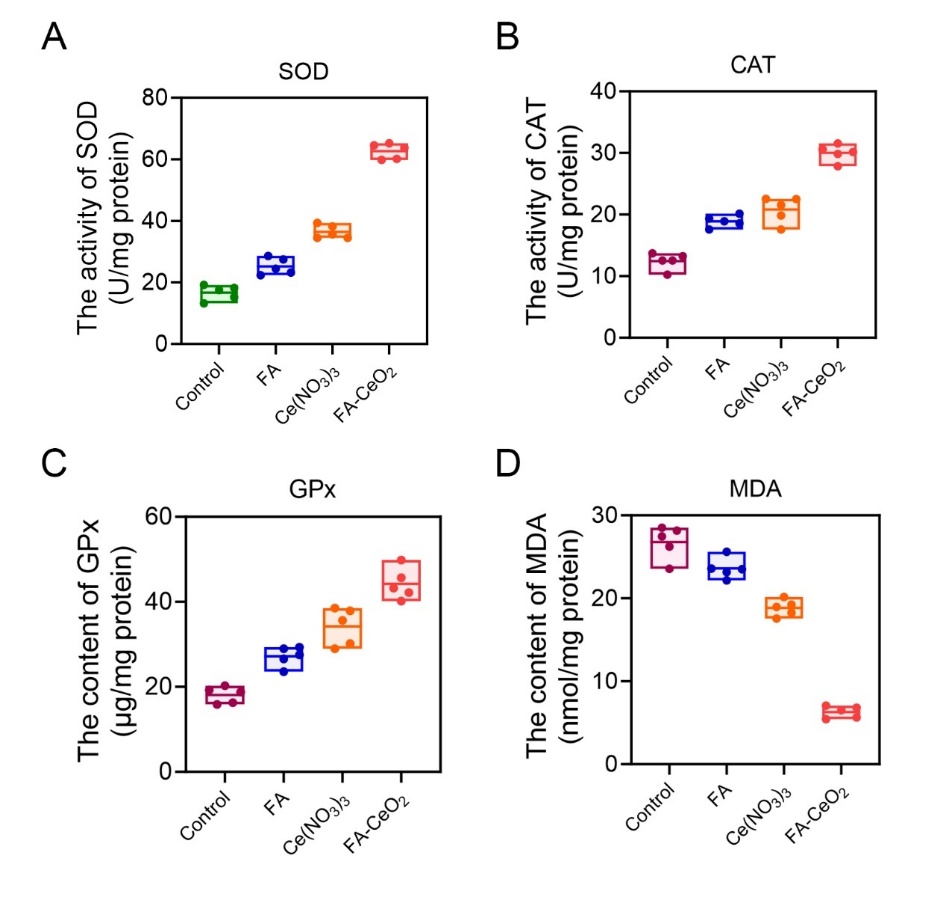


**Figure S17.** Quantification of SOD, CAT, GPx, and MDA expression levels in large-scale and diabetic chronic wound tissue after FA-CeO_2_ treatment, measured via assay kits (n = 5).


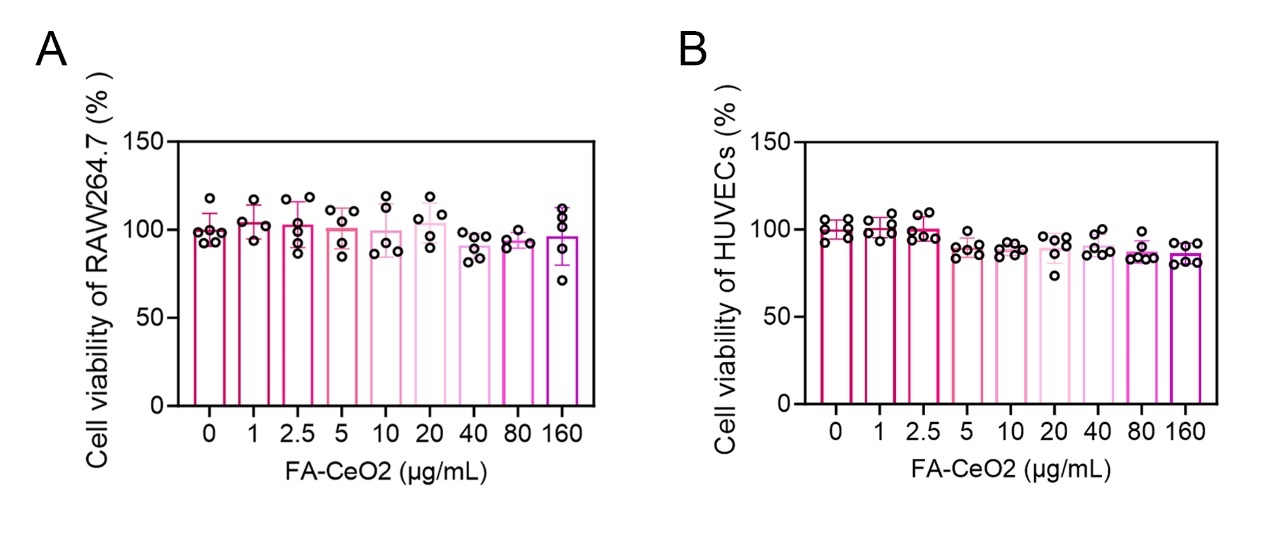


**Figure S18.** Cytocompatibility assessment of FA-CeO_2_ in RAW264.7 **(A)** and HUVECs **(B)** cells treated with various concentrations (n = 6).


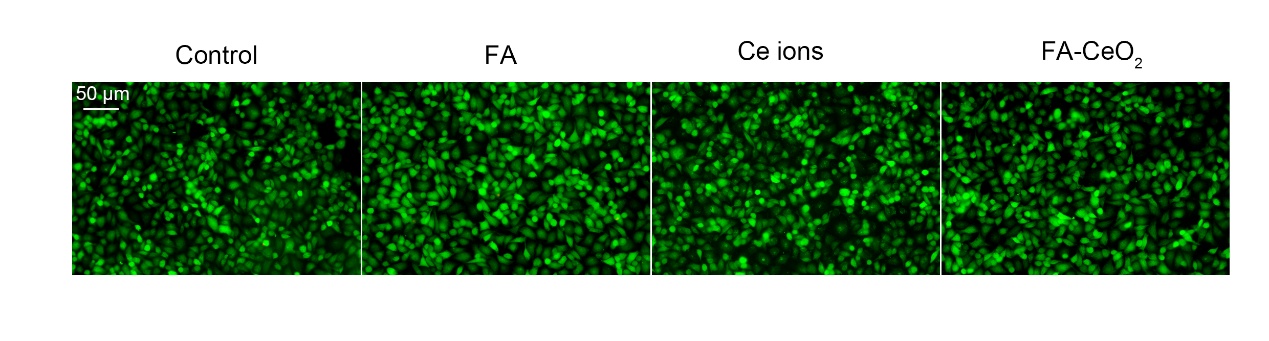


**Figure S19.** Fluorescence images of calcein AM and PI-stained HUVECs cells exposed to FA, Ce ions, and FA-CeO_2_. Scale bar = 50 μm.


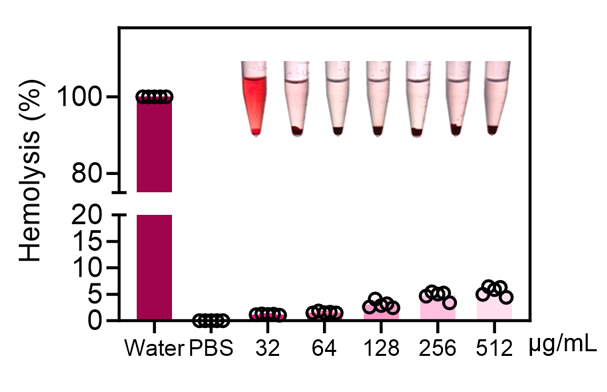


**Figure S20.** The hemolysis test result of FA-CeO_2_ across tested concentrations (n = 5).


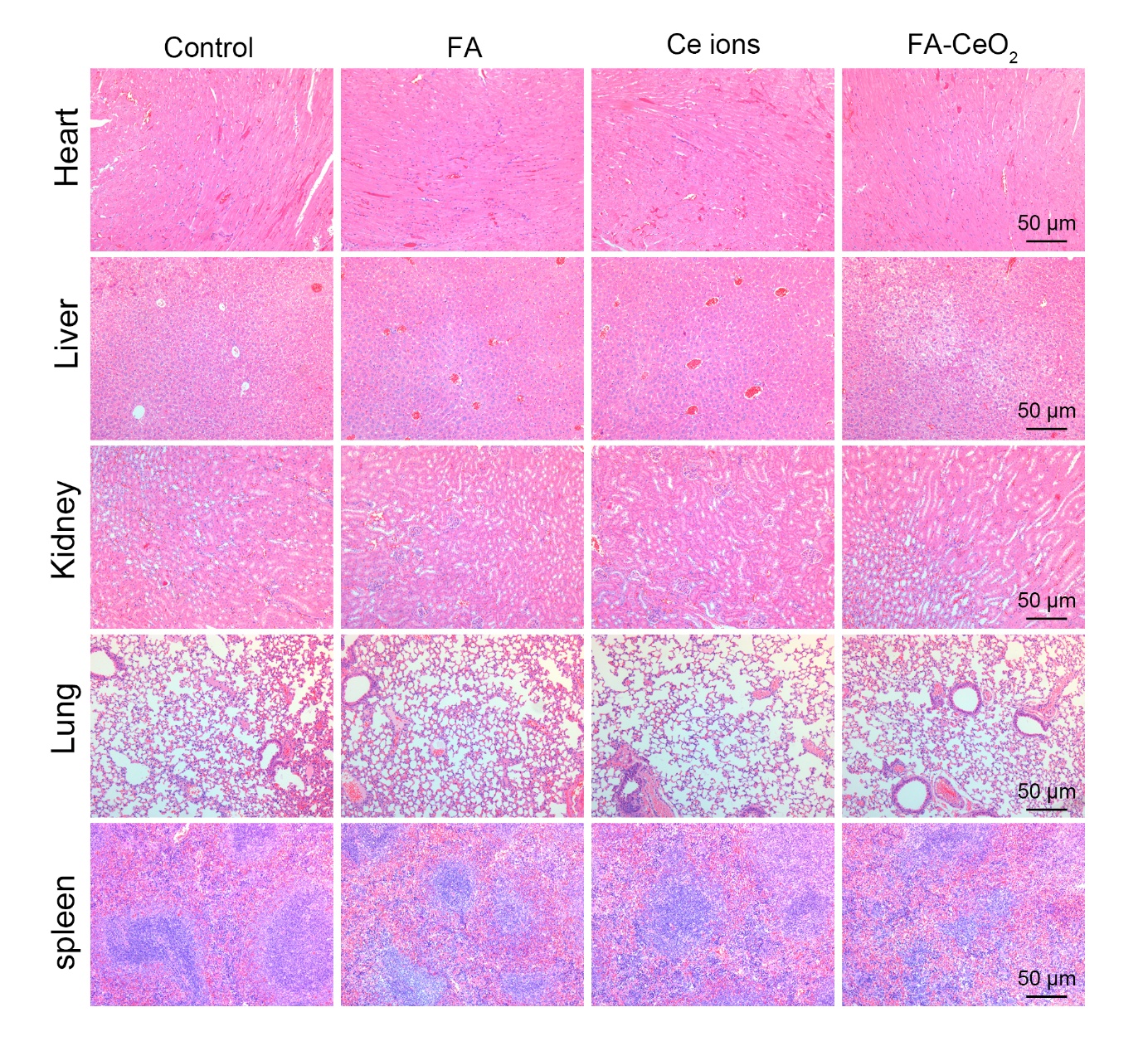


**Figure S21.** H&E-stained sections of significant organs from PBS-, FA-, Ce ions-, and FA-CeO₂-treated mice. Scale bar: 50 μm


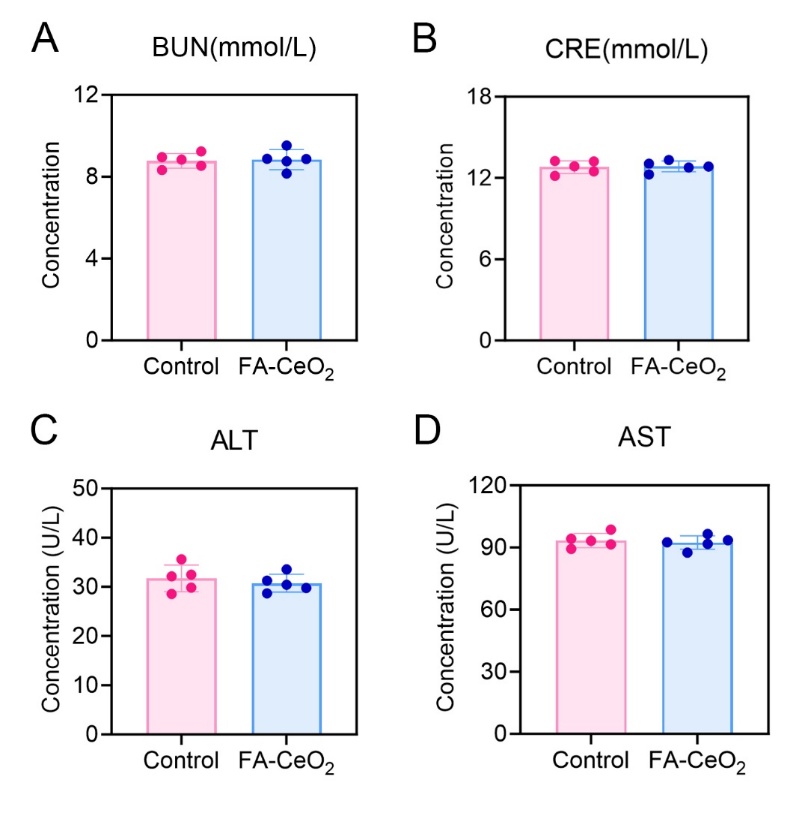


**Figure S22**. **a, d)** Blood biochemistry results (BUN, CRE, ALT, AST) in control and FA-CeO₂-treated mice (n = 5). BUN: Blood Urea Nitrogen, CRE: Creatinine, ALT: Alanine Aminotransferase, AST: Aspartate Aminotransferase.

1. **Supplementary Table.**

**Table S1: The primer sequences for the relative real-time PCR assay.**

| **Primer** | **Nucleotide Sequence (5’-3’)** |
| --- | --- |
| mCD31-R | GTCCTGCTCCGTCTCGGGCA |
| mCD31-F | TGGGCTTCGAGAGCATTTCG |
| mTGF-β-F | GCGTGCTAATGGTGGACCGC |
| mTGF-β-R | GCCAGGAATTGTTGCTATAT |
| mVEGF-F | TCCACCATGCCAAGTGGTCC |
| mVEGF-R | GCCTGCACAGCGCATCAGCG |
| mE-cadherin-F | CTGACAAACCCCCCGTTGGC |
| mE-cadherin-R | AGACCGGCTGGGTAAACTCT |
| mCollagen I-F | GCTGACCTTCATTGACATGG |
| mCollagen I-R | TTCTGGCCGGCCACTCCTGG |
| mMMP1-F | TCGTGACAATTCTCCCTTTG |
| mMMP1-R | GGGTACATCAAAGCCCCGAT |
| mNrf2-F | GTCACTGTGTAAAGCTTTCA |
| mNrf2-R | CCGTTCTGTTTGACACTTCC |
| mNOX1-F | ATCATCTGCTTAGGGATCCA |
| mNOX1-R | CCTTTCAAATATATAAAAAG |
| mHO-1-F | GGAGATAGAGCGCAACAAGC |
| mHO-1-R | GCGTGGGCCACCAGCAGCTC |
| mGPX4-F | TGACGTAAACTACACTCAGC |
| mGPX4-R | CATTTCCACAGTGGGTGGGC |
| mSOD1-F | AGGAAAGTAATGGACCAGTG |
| mSOD1-R | TCCTTTGGCCCACCGTGTTT |
| mGAPDH-F | CATCACTGCCACCCAGAAGACTG |
| mGAPDH-R | ATGCCAGTGAGCTTCCCGTTCAG |
| hCD31-F | TGTCAAGCTAGGATCATTTC |
| hCD31-R | CATGGCCATGACTGAGTACA |
| hVEGF-F | GAGGAGGGCAGAATCATCAC |
| hVEGF-R | CTCAGTGGGCACACACTCCA |
| h-TGF-β-F | CGGCGGGCCCGGCGGCCAGG |
| h-TGF-β-R | TCTGGGATCTTGGAAAGATC |
| hE-cadherin-F | CATCCAACGGGAATGCAGTT |
| hE-cadherin-R | TGAGGATGGTGTAAGCGATG |
| hCollagen I-F | CGATATAGATGGTGCTGTGA |
| hCollagen I-R | ATTGTGGTACGGCCTGGGTT |
| hMMP1-F | GAAAGCCTTCCAACTCTGGA |
| hMMP1-R | TTGGTCCACCTTTCATCTTC |
| hGAPDH-F | GTCTCCTCTGACTTCAACAGCG |
| hGAPDH-R | ACCACCCTGTTGCTGTAGCCAA |

m means Mouse, h means Human.
